# Drug Discovery Using Chemical Systems Biology: Identification of the Protein-Ligand Binding Network To Explain the Side Effects of CETP Inhibitors

**DOI:** 10.1371/journal.pcbi.1000387

**Published:** 2009-05-15

**Authors:** Li Xie, Jerry Li, Lei Xie, Philip E. Bourne

**Affiliations:** 1 Skaggs School of Pharmacy and Pharmaceutical Sciences, University of California San Diego, La Jolla, California, United States of America; 2 Torrey Pines High School, San Diego, California, United States of America; 3 San Diego Supercomputer Center, University of California San Diego, La Jolla, California, United States of America; National Cancer Institute, United States of America and Tel Aviv University, Israel

## Abstract

Systematic identification of protein-drug interaction networks is crucial to correlate complex modes of drug action to clinical indications. We introduce a novel computational strategy to identify protein-ligand binding profiles on a genome-wide scale and apply it to elucidating the molecular mechanisms associated with the adverse drug effects of Cholesteryl Ester Transfer Protein (CETP) inhibitors. CETP inhibitors are a new class of preventive therapies for the treatment of cardiovascular disease. However, clinical studies indicated that one CETP inhibitor, Torcetrapib, has deadly off-target effects as a result of hypertension, and hence it has been withdrawn from phase III clinical trials. We have identified a panel of off-targets for Torcetrapib and other CETP inhibitors from the human structural genome and map those targets to biological pathways via the literature. The predicted protein-ligand network is consistent with experimental results from multiple sources and reveals that the side-effect of CETP inhibitors is modulated through the combinatorial control of multiple interconnected pathways. Given that combinatorial control is a common phenomenon observed in many biological processes, our findings suggest that adverse drug effects might be minimized by fine-tuning multiple off-target interactions using single or multiple therapies. This work extends the scope of chemogenomics approaches and exemplifies the role that systems biology has in the future of drug discovery.

## Author Summary

Both the cost to launch a new drug and the attrition rate during the late stage of the drug discovery and development process are increasing. Torcetrapib is a case in point, having been withdrawn from phase III clinical trials after 15 years of development and an estimated cost of US $800 M. Torcetrapib represents a new class of therapies for the treatment of cardiovascular disease; however, clinical studies indicated that Torcetrapib has deadly side-effects as a result of hypertension. To understand the origins of these adverse drug reactions from Torcetrapib and other related drugs undergoing clinical trials, we introduce a systematic strategy to identify off-targets in the human structural proteome and investigate the roles of these off-targets in impacting human physiology and pathology using biochemical pathway analysis. Our findings suggest that potential side-effects of a new drug can be identified at an early stage of the development cycle and be minimized by fine-tuning multiple off-target interactions. The hope is that this can reduce both the cost of drug development and the mortality rates during clinical trials.

## Introduction

Identification of protein-ligand interaction networks on a proteome-wide scale is crucial to address a wide range of biological problems such as correlating molecular functions to physiological processes and designing safe and efficient therapeutics [1]. Recent protein-ligand interaction studies have revealed that protein targets involved in entirely different pharmacology can bind similar small molecule drugs [2]–[4]. Large scale mapping of polypharmacology interactions indicates that drug promiscuity is a common phenomenon across the proteome [5]. It has been found that approximately 35% of known drugs or leads were active against more than one target. Moreover, a significant number of promiscuous compounds (approximately 25%) have observed activity in completely different gene families. Such drug promiscuity presents both opportunities and challenges for modern drug discovery. On one hand, it is possible to develop high-efficacy drugs by inhibiting multiple targets [6] or to reposition existing drugs to treat different diseases [7],[8]; on the other hand, the off-target effect may result in adverse drug reactions that account for around one-third of drug failures during development [9]. As a result, there is increasing interest in the identification of multiple targets associated with a phenotype [6] and in developing combinatorial therapies to boost clinical efficacy [10]. Chemogenomics has emerged as a new discipline to systematically establish target relationships based on the structural and biological similarity of their ligands [3], [11]–[18]. However, the success of chemogenomics depends on the availability of bioactivity data for the receptors and their associated ligands. For new drug targets, such data are either insufficient or unavailable. Further, the adverse drug reaction may involve receptors that are not well characterized. Complementary to chemogenomics methods, we have developed a chemical systems biology approach to identifying off-target binding networks through their ligand binding sites. The method requires 3D-structure information for the protein but not the ligand, thereby extending the scope of existing chemogenomics approaches. Moreover, the identified off-target binding network is integrated with the reconstructed biological pathways so that the effect of the drug on the biological system can be understood at the system level. In brief (see Methods for further details), our chemical systems biology approach proceeds as follows: 1) The ligand binding site of the primary target is extracted or predicted from a 3D experimental structure or homology model and characterized by a geometric potential [19]. 2) Off-target proteins with a similar ligand binding site to the primary target are identified across the human structural genome using a Sequence Order Independent Profile-Profile Alignment (SOIPPA) [20]. The atomic details of the interactions between the drug and the putative off-targets from step 2 are characterized using protein-ligand docking methods. Based on a normalized docking score the high-ranking off-targets are further investigated. 4) The identified panel of off-targets is subject to structural and functional cluster analysis and incorporated into a network that includes multiple metabolic, signal transduction, and gene regulation pathways. The first and second steps have been implemented in the software package SMAP, available from http://funsite.sdsc.edu.

In this paper, we apply this strategy to identify and analyze a panel of unknown off-targets for Cholesteryl Ester Transfer Protein (CETP) inhibitors. CETP inhibitors represent a new preventive therapy for cardiovascular disease through raising HDL cholesterol. However, clinical studies have revealed that one of the CETP inhibitors, Torcetrapib, has deadly off-target effects as a result of hypertension [21]–[25] and consequently was withdrawn from phase III clinical trial. In contrast to Torcetrapib, another CETP inhibitor JTT-705 does not have unwanted side-effects that increases blood pressure [25]. In addition, JTT-705 is able to block cell proliferation and angiogenesis through Ras and P38 kinase pathways [26]. As will be shown, the multiple off-targets of these CETP inhibitors identified here are involved in both positive and negative control of stress regulation and immune response through an interconnected metabolic, signal transduction and gene regulation network. Our predictions are strongly correlated to the observed clinical and *in vitro* observations, providing a molecular explanation for the difference in side-effect profiles of these two CETP inhibitors. These findings suggest that adverse drug reactions might be modulated by the fine-tuning of the off-target binding network and exemplify the role of systems biology in the future of drug discovery.

## Results

### CETP off-target binding network computed for the human structural genome

The ligand binding site of CETP (PDB id: 2OBD) is assumed to be a long tunnel interacting with two cholesteryl oleates (2OB) and two 1,2-dioleoyl-Sn-glycero-3-phosphocholines (PCW) molecules in the native state (Fig. S1), however, the exact location of inhibitor binding is unknown. Docking studies using the software Surflex [27], eHits [28] and AutoDock [29] indicate that the CETP inhibitors are able to bind to all four sites, with a slight preference for the pocket occupied by PCW. Thus, all four sites were used to search for the off-target binding sites of CETP inhibitors.

**Figure 1:**
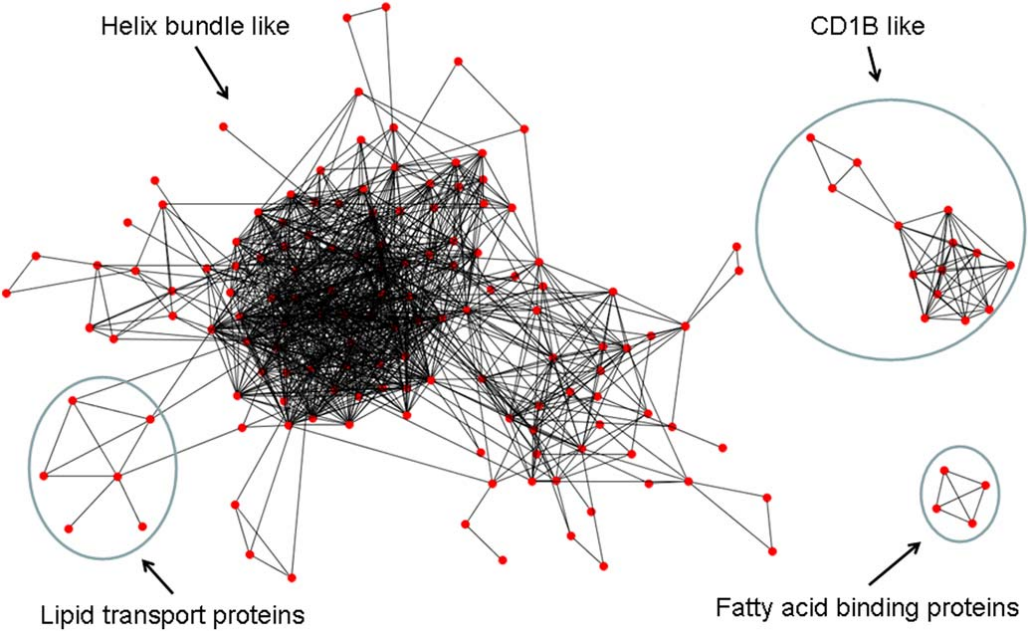
The three largest clusters of the off-target network formed from their global structural similarities. Each node in the graph represents one off-target as found in supplemental material Table S1. Two nodes are connected by an edge if their global structures are similar (measured by a CE [104] z-score larger than 4.0).

Although only approximately 15% of human proteins have known 3D structures deposited in the Protein Data Bank (PDB) [30] , the structural coverage of the human proteome increases to 57% if homologous proteins are included (e-value less than 1.0e-3 and aligned sequence lengths greater than 30 residues using a Blast [31] search). The structural coverage is reduced to around 40% if the aligned length is greater than 120 residues (Fig. S2). After removing structures with redundant sequences (sequence identity = 100%), 5,985 structures and models from the PDB were selected for off-target search by SMAP. Besides bactericidal/permeability increasing protein (PDB Id: 1ewf) that is classified in the same fold and Pfam [32] family as CETP (FATCAT [33] p-value = 1.26e-11, RMSD = 4.53), 273 off-fold structures are found with similar binding sites to CETP (SMAP p-value less than 1.0e-3). Reverse virtual screening of the 273 structures against JTT-705, the smallest CETP inhibitor, was carried out with Surflex [27] and eHits [28] (see Methods) to detect the binding capability of these proteins. To reduce the impact of protein flexibility, the complex structure, whenever available in PDB, is used for docking. Proteins that have steric crashes with JTT-705 were removed from the list and a panel of CETP off-targets consisting of 204 structures was constructed for further study as shown in Table S1. The majority of these off-targets have binding sites that match to one of the two sites that are adjacent to PCW in CETP. Excluding cytochrome P450s that bind drugs promiscuously, most of the putative off-targets are involved in lipid/fatty acid transport or binding, signal transduction pathways and immune response. Based on both SMAP p-values and docking scores (p-value<1.0e-3, Surflex score>3.50 and eHiTs score<−4.50), six classes of structure were consistently found at the top of the list: CD1B like antigen recognition domains (CD1B); nuclear hormone receptor ligand binding domains (NR); lipid transport proteins (LPTP); fatty acid binding proteins (FABP); EF hand-like calcium binding proteins (EF); and heme binding proteins (HEME). The first four classes of proteins are able to bind cognate ligands similar to those that bind to CETP, such as fatty acids, lipoproteins, and lipids [34]. Although these putative off-targets do not have detectable global structural similarities to CETP according to their CE Z-scores (Fig. S3), they have local structural similarity and are related to each other, forming an interconnected off-target network. As shown in Fig. 1, 76% of the putative off-targets (154/204) form the three largest clusters. The largest helix bundle cluster includes NR, EF, HEME and other proteins (Fig. S4). In this paper, we focus on the six selected classes of proteins and demonstrate how they correlate to the clinical findings. Other putative off-targets are subject to on-going computational and experimental studies.

### Structural characterization of CETP off-targets

Most of the predicted ligand binding sites of CD1B, LPTP, and FABP have a similar topology to that of CETP. The drug molecule binds to a cavity formed by anti-parallel beta-sheets and capped by other structural components such as a helix. The others, NR, EF, and HEME all have alpha-helical architectures that are completely different from the secondary structure surrounding the binding site of CETP. These differences illustrate the necessity of tools like SMAP that can find local structural similarities even when global similarity is non-existent. From a functional perspective, it is not surprising that lipid binding proteins act as off-targets for CETP inhibitors since they are required to bind similar cognate hydrophobic ligands such as PCW. It is noteworthy that glycolipid transfer protein, one of the lipid binding proteins, has significant structural similarity to nuclear hormone receptors. For example, the FATCAT [33] p-value is 1.77e-3 when comparing one glycolipid transfer protein (PDB id: 1TFJ) with that of retinoid X receptor (PDB id: 1YOW), but the RMSD is 9.82 Å for a rigid superimposition. However, if the components of these structures are allowed to twist, the RMSD drops to 2.57 Å when the helices surrounding the binding site are well aligned (Fig. S5). The structural similarity between glycolipid transfer protein and other all-helical proteins increases confidence in our result that the lipid-activated nuclear receptor (NR) is one of the major off-targets of CETP inhibitors.

### Functional correlation of CETP with off-targets

We searched for possible functional correlations between CETP and the putative off-targets using the iHOP [35] literature network (http://www.ihop-net.org/UniPub/iHOP/in?dbrefs_1=NCBI_LOCUSLINK__ID|1071). Several top-ranked off-targets appear in the same sentences with each other more than 3 times in the literature. They include phospholipid transfer proteins, nuclear receptors, including PPAR, major histocompatibility complex class II that is similar to CD1B, apolipoprotein A-1, and angiotension I converting enzyme.

The functional similarity between CETP and the off-targets is further quantitatively measured using gene ontology (GO) relationships found with the FunSimMat web server [36] (http://funsimmat.bioinf.mpi-inf.mpg.de/index.php). From 204 off-targets, 148 structures had annotated GO terms and 94 structures had detectable similarities with a Resnik score [37] larger than 0.0. Among these 94 structures, lipid transport/binding proteins, CD1B, and nuclear hormone receptors were ranked top, followed by globin-like, EF hand-like and other proteins (Table S2).

### Binding affinity similarity between CETP and off-targets

To further support our off-target predictions we conducted docking studies on CETP and the identified off-targets, which also provides insights into the molecular mechanisms of off-target binding. It has been established that the binding affinity calculated from docking programs is not necessarily reliable [38]–[40]. When using an energy-based scoring function, the errors come predominantly from the inaccurate parameterization of the individual energy terms. We find that the docking scores for CETP and its putative off-targets are linearly dependent on the number of carbon atoms on the docked molecules because the hydrophobic term dominates the scoring (Fig. S6). Based on this observation we developed a procedure to minimize the systematic error in the scoring function. Rather than considering the raw docking score we used the z-score to represent the relative binding affinity. The z-score is derived from a large number of random drug-like molecules and is dependent on both the number of carbon atoms in the ligand and the nature of the protein binding site. A large negative z-score indicates a high probability of true binding. Based on this procedure, the normalized docking scores (NDS) of the six classes of off-targets are listed in Table 1. These data indicate that binding of CETP inhibitors to putative off-targets is indeed statistically significant. Furthermore, the vector distance of the carbon atom size dependent average docking score for CETP and the majority of off-targets is less than 1.0 (Table S3). This implies that the ligands are able to bind to CETP and to the off-targets with similar binding affinities, since their predicted binding affinity differences are less than 1.0, which is the standard deviation of docking scores (see Methods). Finally, the correlation of ligand binding profiles between CETP and its off-targets [4] are relatively high (Table S3 and Fig. S7).

**Table 1:** Binding site volumes and normalized docking scores (NDS) of CETP inhibitors for CETP and six classes of putative off-targets.

Target Class	Protein	PDB ID	Binding Site Volume (Å^3^)	Normalized Docking Score		
				Torcetrapib	Anacetrapib	JTT-705
	CETP	2OBD$	1084.2	−5.6024^*^	−4.6705^*^	**−1.9644#**
NR	Retinoid X receptor (agonist)	1YOW$	1420.5	−5.5803^*^	−4.1922^*^	**−0.9344#**
	PPARδ (agonist)	1Y0S$	1313.2	−3.8703^*^	−3.8384^*^	**−1.5662#**
	PPARα (agonist)	2P54$	1059.4	−4.0828^*^	**6.6785**	−3.0660^*^
	PPARα (antagonist)	1KKQ$	1012.6	−3.8847^*^	−3.8554^*^	**−0.9725#**
	PPARγ (agonist)	1ZEO$	726.5	−3.9838^*^	**6.0096**	−2.0316^*^
	LXRα (agonist)	2ACL$	1155.0	**5.7793**	**6.3052**	**−0.6900#**
	LXRβ (agonist)	1UPV	1553.5	**5.0882**	**5.5450**	**−1.7543#**
	Vitamin D receptor (agonist)	1IE8$	879.7	**5.7622**	**6.1759**	**−1.1761#**
	Vitamin D receptor (antagonist)	2ZMH$	1055.8	**5.1326**	−3.2234^*^	**−0.8868#**
	Glucocorticoid receptor (agonist)	1P93$	819.0	**5.5504**	**6.1432**	−2.0131^*^
	Glucocorticoid receptor (antagonist)	1NHZ$	990.5	−2.1235^*^	−3.2125^*^	**−1.1673#**
LPTP	Glycolipid transfer protein	1TFJ$	987.4	**−0.9839#**	−2.1587^*^	**−1.3249#**
	Phosphatidylcholine transfer protein	1LN1	1860.1	−7.3050^*^	−9.1032^*^	**−1.0794#**
	Phosphatidylinositol transfer protein	2A1L^*^	2271.7	−4.0881^*^	−6.0708^*^	**−1.7366#**
	GM-2 activator	2AG9	955.0	−4.0254^*^	−3.8265^*^	−3.6934^*^
FABP	Fatty acid binding protein	2NNQ^*^	743.3	**3.3521**	**6.8334**	−2.3356^*^
CD1B	CD1B receptor	1GZP	1056.4	−2.0899^*^	−6.1531^*^	**−1.3424#**
EF	Troponin C	1DTL	1992.0	−3.7771^*^	−3.6403^*^	−2.9955^*^
HEME	Cytochrome complex	1PP9	2963.0	−3.8702^*^	−7.0825^*^	−2.2745^*^
	Human cytoglobin	1V5H	1022.2	−3.4827^*^	**−1.8246#**	−2.3848^*^

The predicted binding affinities are grouped with different symbols: * represents strong binding affinity (NDS<−2.0).

# relatively strong (0.0>NDS>−2.0), and others represent less likely binding due to the steric crashes between the ligand and the protein (NDS>0.0), respectively.

$ indicates that Cystine appears in the binding site and may form disulfide bonds with JTT705.

Importantly, the binding profiles for the three CETP inhibitors (Torcetrapib, Anacetrapib, and JTT-705) are different from each other across the panel of off-targets. JTT-705 is the most promiscuous inhibitor. In contrast, Torcetrapib failed to dock into some of the off-targets, and Anacetrapib is suitable to be docked into the least number of off-targets. The difference between their off-target binding profiles can be partly explained by their different complexity [41] and sizes. The molecular volumes of JTT-705, Torcetrapib and Anacetrapib are 407.31, 498.42, and 527.28 Å^3^, respectively. As shown in Table 1 the estimated volume of the off-target binding pockets varies greatly. Thus, the smallest ligand, JTT-705, can be accommodated in all of these pockets, but the larger-sized Torcetrapib and Anacetrapib are difficult to fit into the smaller sized pockets. It could be argued that the failure in docking Torcetrapib and Anacetrapib into the smaller sized pockets is because the induce fit of the receptor is not explicitly modeled. However, for most of the NRs, both antagonist and agonist conformations are tested. Thus it is less likely that the unfitness of Torcetrapib and Anacetrapib for some of the off-targets is a result of not specifically considering induced fit in the docking calculation. The different off-target binding profiles of these CETP inhibitors have significant implications for the observed side-effects, as discussed subsequently.

### Incorporation of the off-target binding network into biological pathways

**Figure 2:**
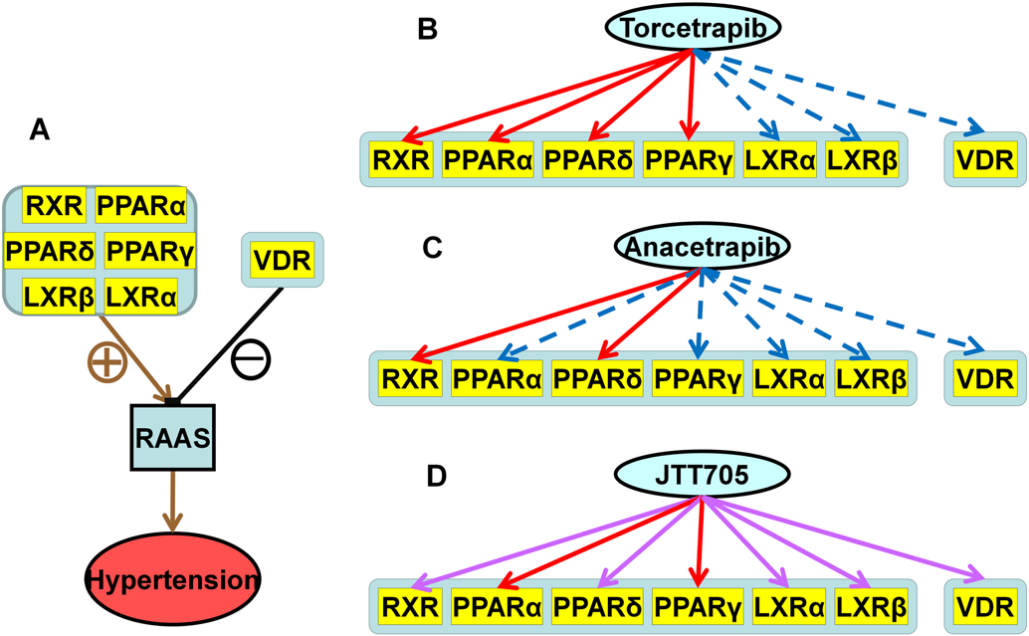
Effects of Torcetrapib, Anacetrapib and JTT-705 in regulating the RAAS system through the combinational control of nuclear hormone receptors. The red, purple, and blue lines between inhibitors and off-targets indicate strong, relatively strong, and weak binding affinities, respectively. The brown and black lines between off-targets and pathways or clinical indications represent positive and negative regulation, respectively. A. Regulation control of nuclear hormone receptors on RAAS system. B. Binding profile of Torcetrapib on nuclear hormone receptors. C. Binding profile of Anacetrapib on nuclear hormone receptors. D. Binding profile of JTT-705 on nuclear hormone receptors.

**Figure 3:**
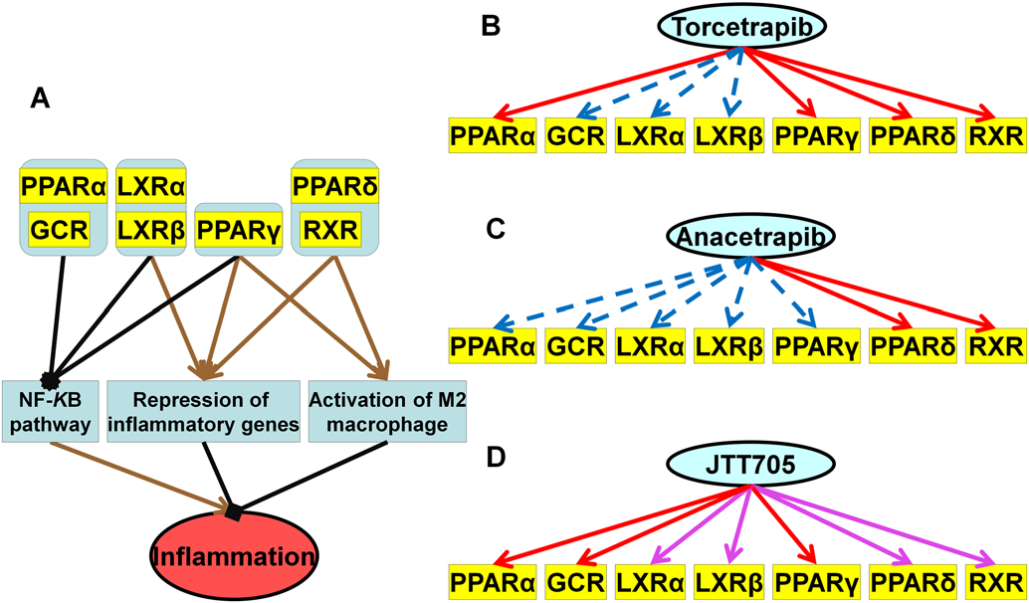
Effects of Torcetrapib, Anacetrapib and JTT-705 on inflammation through combinational control of nuclear hormone receptors. The color and line schema are the same as those in Fig. 2. A. Regulation control of nuclear hormone receptors on inflammatory system. B. Binding profiles of Torcetrapib on nuclear hormone receptors. C. Binding profile of Anacetrapib on nuclear hormone receptors. D. Binding profile of JTT-705 on nuclear hormone receptors.

**Figure 4:**
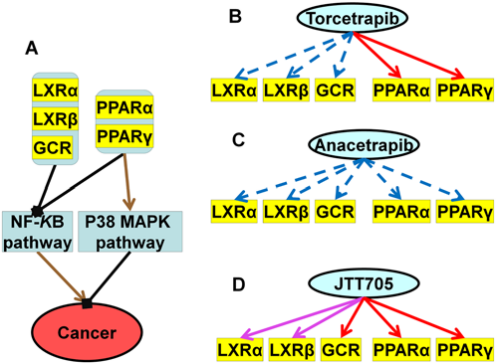
Effects of Torcetrapib, Anacetrapib and JTT-705 on cancer through combinational control of nuclear hormone receptors. The color and line schema are the same as those in Fig. 2. A. Regulation control of nuclear hormone receptors on cancer system. B. Binding profiles of Torcetrapib on nuclear hormone receptors. C. Binding profile of Anacetrapib on nuclear hormone receptors. D. Binding profile of JTT-705 on nuclear hormone receptors.

By incorporating the predicted off-targets into biological pathways it is possible for us to correlate the predicted off-target interactions with the observed pleotropic effects of Torcetrapib, Anacetrapib and JTT-705. Among them, the negative effect of Torcetrapib on blood pressure in phase III clinical trials could be deduced. Also deducible was an explanation for the increased death from infection and cancer [21]. Conversely, JTT-705 has gotten encouraging safety results from phase II clinical trials and no side-effects of hypertension have been observed thus far. Similar positive results are observed for Anacetrapib during phase I clinical trials. It should be noted that at this time that JTT-705 and Anacetrapib are in clinical trials involving only a small number of patients during short term studies. Results from long term studies are needed to confirm the absence of negative effects for these two drugs. In addition, JTT-705 is found to be able to block cell proliferation and angiogenesis through Ras and P38 kinase pathways [26]. To illustrate these findings, using a survey of the literature, we constructed a hierarchical biological network that connects drugs, off-targets, pathways and clinical observations. Using this network we could explore the implications of administering CETP inhibitors on different pathways through their interactions with corresponding off-targets (Fig. S8). The network consists of several interconnected metabolic, signal transduction, and gene regulation pathways. Each component of the network is separately shown in Fig. 2, Fig. 3, Fig. 4, and Fig. S9, and is discussed in detail in the following sections. It is notable that several predicted off-targets, especially the nuclear hormone receptors, are essential components in the network, involved in both positive and negative controls of several cellular systems. Nuclear hormone receptors are known as lipid-activated transcription factors that play key roles in lipid metabolism, inflammatory processes and the hormone system. The regulatory controls of our predicted nuclear hormone receptors are on pathways involved in hypertension, inflammation and cancer development. Torcetrapib, Anacetrapib and JTT705 showed different binding affinities to these receptors and thus different clinical outcomes resulting from the combinational responses of these receptors in related pathways.

#### Combinatorial control of nuclear hormone receptors in hypertension

As shown in Fig. 2, the effects of the three CETP inhibitors on blood pressure can be explained through their influence on the Renin-Angiotension-Aldosterone System (RAAS), the main system for blood pressure regulation. When the RAAS system is too active, blood pressure becomes dangerously high. Several nuclear receptors highly ranked in our off-target list are involved in the regulation of this system, including proxisome proliferator-activated receptor (PPAR), retinoid X receptor (RXR), liver X receptor (LXR) and Vitamin D receptor (VDR) [42]. As positive regulators, activation of PPAR, RXR and LXR increases gene expression of angiotensinogenase and then up-regulates RAAS, resulting in high blood pressure and increased aldosterone secretion. In contrast, VDR has a negative control on RAAS. Activation of VDR will balance the up-regulation effect of PPAR, RXR and LXR on blood pressure.

According to the normalized docking scores (Table 1), Torcetrapib, Anacetrapib and JTT-705 show distinctly different binding profiles to these nuclear receptors, consistent with their differing involvement in hypertension. To avoid inaccuracy in the docking calculation, three different categories (strong, relatively strong and weak) were used to estimate the binding affinity, instead of direct comparison of individual docking scores. It is noteworthy that the weak binding (large positive normalized docking score) is due mainly to the steric crashes between the CETP inhibitors and the receptor. As a result the inhibitors cannot fit into the binding pockets of these receptors. Interaction between the three CETP inhibitors and nuclear receptors are shown in Fig. 2 with different colors illustrating the type of interaction. When the three CETP inhibitors are docked as agonists to these nuclear hormone receptors the stronger binding affinity to the panel of positive regulators (PPAR, LXR and RXR) indicates stronger up-regulation of RAAS and higher risk of hypertension; stronger binding affinity to the negative regulator VDR implies a lower risk of hypertension. It is clearly shown in Fig. 2 that JTT-705 has relatively strong binding affinity not only to the positive regulators but also as an agonist to the negative regulator, implying the ability of JTT-705 to exhibit a balanced positive/negative control over RAAS and consequently a lesser chance to cause hypertension. In contrast to JTT-705, Torcetrapib binds more strongly to the active conformations of the positive regulators and leads to increased blood pressure through up-regulation of RAAS. Limited by its larger size, Anacetrapib can only bind to RXR and PPARδ in their active conformations and PPARα and VDR in their inactive conformations (listed in Table 1), even though it is in the same structural class as Torcetrapib. Thus, Anacetrapib has less effect on both the positive and negative control of blood pressure and its negative effect on blood pressure regulation may be less than Torcetrapib. Even though the detailed mechanism for the side-effect of hypertension caused by Torcetrapib is still unknown and the different binding profiles of the three CETP inhibitors needs experimental verification, our observations are consistent with the current clinic trial data from the three CETP inhibitors and the predicted off-targets provides information for future use in drug optimization.

#### Combinatorial control of nuclear hormone receptors in inflammation

The effects of CETP inhibitors on inflammation are shown in Fig. 3. Activation of nuclear hormone receptors such as PPAR, LXR, GCR and RXR regulates gene expression associated with inflammation through different mechanisms [34],[43] and consequently reduces the inflammatory response. For example, NF-κB plays a key role in regulating the immune response to infection. PPARα/γ, LXRα/β and GCR can block the NF-κB pathway by directly binding to AP1 and NF-κB [44]–[48], acting downstream of NF-κB binding to DNA [49], or by competing for limited amounts of co-activators [50]. There are other examples to show the PPAR induced trans-repression of inflammatory response genes [51],[52]. PPARγ/δ can also function as transcriptional regulators of monocyte phenotypic differentiation by promoting expression of target genes involved in M2 macrophage function thereby activating M2 macrophage so as to generate anti-inflammatory products [53]–[55]. Thus, the overall picture of activation of these nuclear hormone receptors involved in inflammatory response suggests that they have interesting anti-inflammatory effects. The binding profiles of Torcetrapib, Anacetrapib and JTT-705 to these nuclear hormone receptors (listed in Table 1 and shown in Fig. 3) indicate that JTT-705 has a broader control over the inflammation system to reduce the inflammatory response.

#### The regulatory effect of nuclear hormone receptors on cancer

NF-κB regulates genes involved in cell proliferation and cell survival and hence is an interesting drug target in cancer treatment. Inhibition of NF-κB can potentially halts tumor progression and eliminate tumors [56],[57]. As discussed above, activation of PPARα/γ, LXRα/β and GCR will block the NF-κB pathway and thus prevent cancer. Of the three CETP inhibitors only JTT-705 is predicted to bind to these receptors and hence have the ability to control cell proliferation and tumor progression (Fig. 4).

**Figure 5:**
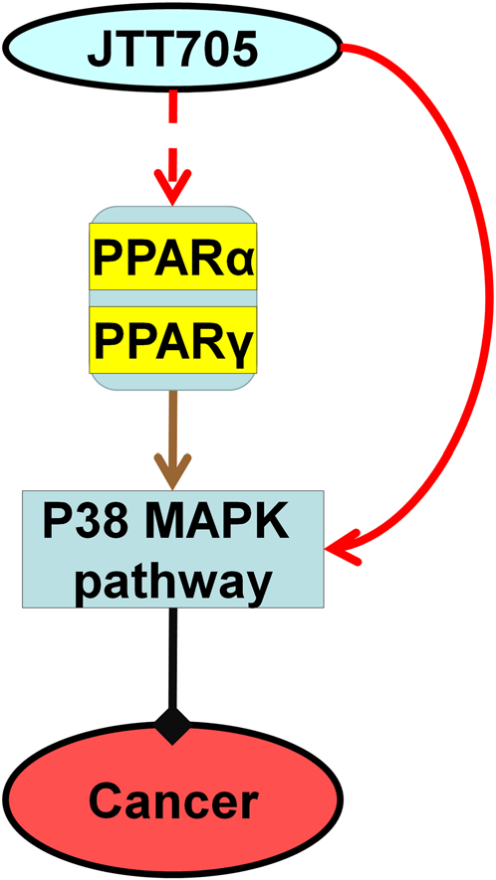
The anti-proliferation effect of JTT-705 through activation of PPARα/γ and the p38 MAPK pathway. Solid lines show relationships established with experimental evidence. Red dash lines show our hypothesis for how JTT-705 induces the activation of the p38 MAPK pathway. Color and line schema are the same as those in Fig. 2.

Recent experiments have shown that PPARα and PPARγ can induce extracellular signal-regulated kinase (Erk) and/or p38 phosphorylation and then activate the MAPK/Erk signaling pathway [58],[59]. This pathway is involved in the action of most nonnuclear oncogenes and participates in cancer development [60]. Interestingly, JTT-705 was shown to block cell proliferation through the activation of the p38 MAPK pathway [26], but the mechanism for how JTT-705 induces p38 MAPK activation is still unclear. Our results suggest a possible hypothesis (Fig. 5). JTT-705 could trigger the p38 MAPK pathway through its interaction with PPARα/γ and thus has the potential to prevent cell proliferation and cancer.

#### Regulatory effects of other identified off-targets

Effects of PPAR and RXR are also regulated by fatty acid binding proteins (FABP) [61]. FABP can function as an intracellular chaperone to transport fatty acids and drugs into the nucleus and directly interact with PPAR [62]. The cooperation between FABP and PPAR will enhance the activities of PPAR in gene transcription regulation [63],[64]. FABP can also interact with hormone-sensitive lipases to potentially modulate their catalytic activity and thereby integrates several signaling networks that control inflammatory response potentially through the JNK/inhibitor of kappa kinase (IKK) and IKK–nuclear factor-κB (NF-κB) pathway [61]. According to the calculated docking scores, only JTT-705 can bind to FABP and further regulate hypertension and inflammation.

Another type of highly ranked off-target, CD1, can also be directly related to the side- effect of infection through its function as an antigen-presenting protein in the immune system. T cells will recognize antigens presented by CD1 proteins and activate a cell-mediated immune response against microbial infections [65]. Docking results show that all three drugs have a strong binding affinity to CD1, suggesting an impact on antimicrobial immunity and host response to infection.

Other putative off-targets such as ubiquinol-cytochrome-c reductases, globin-like proteins, EF hand-like calcium binding proteins (EFs), and LPTP are also directly or indirectly associated with hypertension, inflammation, and/or cancer. Recent studies suggest that ubiquinol-cytochrome-c reductase expression is indirectly regulated by steroid hormones in response to hypertension [66]. Further, as one of the key protein components in the Q-cycle [67],[68] it contributes to the regulation of cell death and repair [69] and may also be related to cancer and infection. It is interesting that hemoglobin has been found in non-hematopoietic organs such as the kidney acting as an anti-oxidative defense agent [70]. It is also involved in the activation of KCl cotransporter activity [71],[72], which may affect the regulation of blood pressure. EFs modulate vascular function through Ca^2+^ homeostasis and nitric oxide. It has also been observed that the lack of S100A1 (an EF protein) expression could lead to hypertension [73]. EFs also have effects on transcription factors. They not only indirectly regulate the activity of transcription factors through their phosphorylation/dephosphorylation in response to Ca^2+^ levels but also directly control the transcriptional activity of the tumor suppressor p53 through interactions with its regulatory sequences [74]. It is not surprising that LPTP is one of CETP's off-targets because they bind to the same or similar cognate ligands and are involved in lipid metabolism. However, the biological functions of phosphatidylinositol/phosphatidylcholine transfer proteins (PITPs) have not been well characterized [75],[76]. They may play a role in dense-core vesicles exocytosis, which regulates heart rate and blood pressure through the release of noradrenaline and adrenaline [77]. Interactions between the three CETP inhibitors and these predicted off-targets show potential additional contributions to the side-effects of hypertension, inflammation and cancer.

In summary, most of the putative off-targets for CETP inhibitors are involved in interconnected lipid metabolism and signaling networks which activate or mediate various biological process such as hypertension, stress regulation [78], immune response [79] and cell death [80]. Our predications are consistent with current clinical studies on all three CETP inhibitors, highlighting the interrelationship of multiple biological processes involved in hypertension, infection and cancer. These results call for further experimental validation.

## Discussion

### Roles of combinatorial control in modulation of side-effects of CETP inhibitors

*In vitro*, *in vivo* and clinical studies indicate that CETP inhibitors exhibit pleotropic effects in humans through the interaction with unknown off-targets. We have identified a panel of proteins that likely bind to CETP inhibitors leading to the observed clinical indications. The putative off-target interactions are consistent with existing experimental data and provide insights into the molecular mechanisms of the side-effect profile of CETP inhibitors. Drug promiscuity depends not only on the similarity of ligand binding pockets in the related proteins but also the complexity of the drug itself [41]. In general, smaller molecules are able to bind more targets. The same trend has been predicted for CETP inhibitors; the smallest JTT-705 is the most promiscuous and the largest, Anacetrapib, is the least promiscuous. However, in contrast to conventional wisdom that implies the more specific the binding the lesser the side-effects, the most promiscuous inhibitor, JTT-705, does not cause the side-effect of hypertension that is observed in the more specific Torcetrapib. Considering the regulation of blood pressure by NRs, it is possible that JTT-705 acts as an antagonist of NRs to down-regulate aldosterone. However, our results suggest that CETP inhibitors prefer binding to the agonist rather than the antagonist conformation of the NR. Experimental evidence also implies that JTT-705 actually activates NR to mediate Ras and p38 kinase pathways [26]. Thus, it is more likely that the side-effect of CETP inhibitors is modulated by a combination of biological controls involved in many physiological processes such as cell proliferation [81], inflammation and hypertension. In other words, JTT-705 is involved in activation of NRs that contribute to both positive and negative controls of aldosterone. Although Torcetrapib is more specific and binds less off-targets than JTT-705, it only activates those NRs that up-regulate RAAS resulting in hypertension. To fully understand how small molecules can modulate physiological or pathological processes through such combinatorial control, it is necessary to simulate the dynamic properties of the biological system. To this end, it is a critical first step to identify all of the putative molecular receptors involved in the biological process and to connect them into a logical integrated protein-ligand interaction network.

### Advantages and limitations of the methodology

The chemical systems biology approach developed here is limited by available protein structures that currently only cover approximately 50% of the human proteome, although the structural coverage of the human proteome will steadily increase with progress in structural genomics [82] and conventional structure determination. As a result, some potential off-targets may be missed because they are not included in the screening. In addition to establishing functional relationships between proteins using their sequences, structures and functional sites, there are significant efforts to relate drug targets to their ligands through chemical genomics analysis [12]. However, the chemical genomics approach is restricted by the availability of bioactivity data. When exploring off-targets that cover the whole human proteome, this limitation becomes obvious since only a small number of target families explored by pharmaceutical companies are in the bioactivity database [5]. Thus our method is complementary to existing chemical genomics approaches. Drug-target networks will be greatly expanded by combining chemical genomics data and a structural genome-wide off-target analysis. Several studies have attempted to extend the target-based method to the domain-based model through similar sequence motifs or global structures [83]. In this study we further expand the scope of the chemical genomics approach beyond sequence and fold similarity by searching for similar ligand binding sites. Hence a ligand binding site-based approach will provide an ever improving way to generate a candidate list of proteins participating in interconnected biochemical pathways and to establish their relationships to biological processes. It is hoped that these approaches will eventually provide the foundation for the *in silico* simulation of the influence of small molecules on biological systems. In the interim it is noted that the analysis of incomplete networks is still invaluable in making new discoveries in biomedicine as exemplified by several recent studies [3],[11].

Besides SMAP used in this study, a number of web servers for ligand binding site search are available, for example, SiteEngine [84], SitesBase [85],[86], CavBase [87]–[89], SuMo [90], PdbSiteScan [91], eF-Site [92],[93], pvSOAR [94], and pevoSOAR [95]. Compared with these servers, SMAP has several distinguishing features making it particularly suitable for identifying off-targets on a structural genome-wide scale. First, SMAP does not require prior knowledge of both the location and the boundary of the ligand binding site. Instead, whole proteins are scanned to find the most similar local patch in the spirit of local sequence alignment such as the Smith-Waterman algorithm [96]. This feature makes SMAP appropriate for practical problems since typically the boundary of the ligand binding site is not clearly defined or depends on the ligand in the complex structure. Second, SMAP integrates geometric, evolutionary and physical information into a unified similarity score akin to a sequence alignment score. However, unlike conventional sequence alignment, the SMAP alignment is sequence order independent; a necessary requirement when comparing local binding sites. Third, because SMAP uses the reduced structure representation, it is not sensitive to structural uncertainty and flexibility. Thus SMAP can be applied to homology models and handle flexible ligand binding sites. Finally, we have developed a probability model to efficiently estimate the statistical significance of the binding site similarity. The model allows us to reliably identify similar ligand binding sites in a high throughput fashion. Despite these advantages of SMAP, it is expected that the best results will come from the combination of different tools as demonstrated by many studies in bioinformatics and molecular modeling.

Despite the success of ligand binding search algorithms in protein function prediction and drug design [2], [4], [20], [87], [88], [95], [97]–[99] currently no algorithm can retrieve all of the binding sites that bind a cognate ligand such as ATP. However, in the context of searching for off-targets of drug molecules, the actual number of false negatives may be limited based on the nature of the drug. False negatives in the ligand binding site search are due mainly to large conformational changes of the ligand and corresponding physical and geometric changes in the binding site. Most existing drugs are designed to selectively inhibit an exquisite target. They are more rigid and less adaptable to the changing environment of the binding site than the cognate ligand. For example, a protein kinase ATP competitive inhibitor is designed to inhibit only the ATP binding site of the protein kinase, not that of other superfamilies such as P-loop hydrolases. On the other hand, although rational drug design may take the same cognate ligand binding site into account, it rarely explores the cross-reactivity between binding sites that are not naturally designed for the same cognate ligand but are able to bind the same drug. Studies by others have shown that the drug binding site can be considered as a negative image of the drug to screen compound database [100] or vice versa to model the drug binding site [101]. Hence ligand binding site similarity search is a valuable tool to identify off-targets that accommodates only the drug molecule but not necessarily all proteins that bind to the same cognate ligand across gene families. In general, the chemical systems biology approach developed in this paper is specific in identifying potential off-targets for drug-like molecules and could be used in concert with experimental design employing *in vitro screening*, *in vivo* screening and clinical trials.

### Implications for drug discovery and development

Even with the current limited structural coverage of the human proteome, our predications are able to provide a testable hypothesis as to the suitability of a lead compound prior to conducting a clinical trial. Thus our findings have implications for drug discovery and development. In contrast to the conventional drug discovery process in which drug leads are optimized to reduce promiscuous binding, the possible combinatorial control of aldosterone regulation by CETP inhibitors suggests that adverse drug effects can be minimized through fine tuning of multiple off-target interactions. Although it is desirable for a drug to bind the primary target in a highly specific way, this is difficult to achieve considering the inherent similarity among protein binding pockets within and across gene families. Moreover, many biological process involve combinatorial control to provide redundancy and homeostasis [102]. In such cases it becomes very difficult to modulate the systems behavior by inhibiting or activating only one single target protein. Thus, a multiple-target approach [6] and combination therapy [10] have been actively pursued to boost clinical efficacy in the treatment of diseases such as cancer and diabetes. However, these combined approaches are rarely systematic with the purposeful intent of developing therapeutics that bind to a primary target to treat the disease, but at the same time are considered to bind to desirable off-targets that modulate side-effects. In some cases this combined goal is achieved serendipitously as would seem to be the case for JTT-705. Instead of using a single molecule, it may be more feasible to use multiple components to treat a disease state and at the same time to reduce drug side-effects. Different from conventional combination therapy where all of components target disease related proteins, here only a subset of the molecules are directly therapeutic, other molecules serve the purpose of reducing side-effects by targeting non-disease related proteins. We speculate that many drugs which failed due to off-target effects can be rescued by this target-off-target combination therapy. For example, it is expected that the side-effect of Torcetrapib can be reduced by introducing molecules that binds to molecular components involved in the negative control of aldosterone regulation. Such therapies can be only rationally designed by exploring the system properties of the biological network.

## Methods

### Binding site similarity search on a genome scale

5,985 structures or models that cover approximately 57% of the human proteome were searched against CETP (PDB id: 2obd) ligand binding sites using the sequence order independent profile-profile alignment (SOIPPA) algorithm [20]. A new statistical model was introduced to the original approach to estimate the significance of the alignment score [103]. In brief, the alignment score for a given alignment length is fitted to an extreme value distribution (EVD):
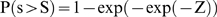
(1)Where:
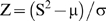
(2)where S is the raw SOIPPA similarity score. μ and σ are fitted to the logarithm of *N*, which is the alignment length between two proteins:
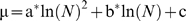
(3)

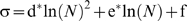
(4)Six parameters a, b, c, d, e, and f are 5.963, −15.523, 21.690, 3.122, −9.449, and 18.252 for the McLachlan similarity matrix used in this study, respectively.

Using this statistical model, 276 off-targets are identified with p-values less than 1.0e-3.

### Reverse screening of the human structural proteome

The putative 276 off-targets are subject to further investigation using more computationally intensive protein-ligand docking. After removing three structures with the same fold as CETP, JTT-705, the smallest CETP inhibitor, is docked to the remaining 273 structures using two commonly used fast docking programs, Surflex 2.1 [27] (default setting) and eHits 6.2 [28] (fastest setting). 69 structures with a Surflex docking score smaller than 0.0 or an eHits score larger than 0.0 are considered to be difficult to fit JTT-705 due to significant steric crashes (and hence the other two inhibitors based on size) and are removed from the putative off-target list. The remaining 204 structures are subject to further investigation using the docking software AutoDock4.0 [29] and other more computationally intense methods as described below.

### Global structure similarity network of off-targets

An all-against-all global structural similarity analysis between the 204 putative off-targets was computed using CE [104]. A graph is constructed with each of the structures as a node. An edge is formed between two nodes if their CE z-score is larger than 4.0 (a superfamily level similarity) [104].

### Volume of the binding pocket

The volume of the binding pocket is computed using the CASTp server [105] (http://sts-fw.bioengr.uic.edu/castp) with default settings.

### Normalized docking score

Drug-like molecules are downloaded from ZINC (http://zinc.docking.org) [106]. From this database, six sets of molecules are randomly selected with a fixed number, 5, 10, 15, 20, 25 and 29 carbon atoms, respectively; each set includes 100 molecules. These molecules are docked to CETP and its putative off-targets using eHiTs [28] and AutoDock4.0 [29]. The correlation of the docking score to the number of carbon atoms is derived from linear regression for each of the protein receptors. From the linear fitting curve, the average docking score for molecules with a certain number of carbon atoms can be estimated.

Based on the fitted average docking score, a normalized docking score *DS* is calculated as a z-score:
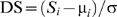
(5)Where *S_i_* is the raw docking score for the molecule with *i* carbon atoms, μ*_i_* is the fitted average docking score for the number of carbon atoms *i*, σ is the standard deviation, which is not dependent on the size of molecules and is approximately 1.0 in all cases.

### Vector distance of the average docking score

The vector distance of the average docking score *D* between CETP and its off-targets is calculated from the average values of the docking scores for randomly selected molecules with fixed numbers of 5, 10, 15, 20, 25 and 29 carbon atoms as follows:
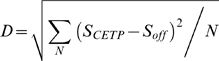
(6)where *S_CETP_* and *S_off_* are the average values of carbon atom size dependent docking scores to CETP and its off-targets, respectively.

### Conclusion

In this case study, we identify a panel of off-targets of CETP inhibitors using a chemical systems biology approach. All of the identified off-targets belong to different protein superfamilies from the primary target, but are structurally and functionally related, being mainly involved in lipid metabolism, immune response and signaling networks. Among them, CD1, nuclear hormone receptors and lipid transport proteins are the most likely off-targets with highly consistent results from multiple resources including functional correlation, ligand binding site similarity, hydrophobic scales, and predicted binding affinities. Moreover, the elucidated off-target effects from these proteins are strongly correlated to clinical and *in vitro* observations. Their combinatorial control of biological process plays a key role in the modulation of the adverse drug effect of CETP inhibitors. This study demonstrates that a chemical systems biology approach, which systematically explores protein-ligand interactions on a genome-wide scale and incorporates them into biological pathways, will provide us with valuable clues as to the molecular basis of cellular function. At the same time, it will help to transform the conventional single-target-single-drug drug discovery process to a new multi-target-multi-molecule paradigm.

## Supporting Information

Figure S1 Four endogenous ligands in the CETP complex structure (PDB id: 2OBD).
(0.09 MB DOC)

Figure S2 Structural coverage of the human proteome vs. alignment length between the protein sequence and the structural template.
(0.06 MB DOC)

Figure S3 CE Z-score distributions of putative off-targets.
(0.06 MB DOC)

Figure S4 Structural clusters of helix-like proteins.
(0.07 MB DOC)

Figure S5 Global structure similarity between glycolipid transport protein (PDB: 1tfj) and nuclear hormone receptor ligand binding domain (PDB: 1yow).
(0.17 MB DOC)

Figure S6 Regression curves of eHiTs score for CETP and its off-targets dependent on the number of carbon atoms for a) 2obd, b) 1yow, c) 1y0s, d) 2p54, e) 1zeo, and f) 1ie8.
(0.38 MB DOC)

Figure S7 Correlation of eHiTS score between CETP and its off-targets binding with random ligands with different sizes. a) 1yow; b) 1y0s; c) 2p54; d) 1zeo; e) 1ie8; f) 1tfj.
(2.04 MB DOC)

Figure S8 Correlation of the off-target interaction network of CETP inhibitors with the clinical indication through interconnected biological pathways.
(0.23 MB DOC)

Figure S9 The different regulation effects of Torcetrapib, Anacetrapib and JTT-705 on hypertension, inflammation and cancer through combinational control of other identified off-targets.
(0.10 MB DOC)

Table S1 Putative off-targets of CETP inhibitors across the human structural genome identified from the off-target pipeline SMAP.
(0.05 MB DOC)

Table S2 GO based similarity between CETP and off-targets.
(0.03 MB DOC)

Table S3 Vector distances and Pearson correlations of carbon atom dependent average eHiTS docking scores between binding pockets of CETP and six classes of off-targets.
(0.05 MB DOC)
