# Supplementary material for: Drug Discovery Using Chemical Systems Biology: Identification of the Protein-Ligand Binding Network To Explain the Side Effects of CETP Inhibitors
Source: PLoS Comput Biol. 2009 May 15;5(5):e1000387. doi: 10.1371/journal.pcbi.1000387 (PMC2676506; doi:10.1371/journal.pcbi.1000387)
Supplement: Figure S1 — Four endogenous ligands in the CETP complex structure (PDB id: 2OBD). (0.09 MB DOC) [file pcbi.1000387.s001.doc]

**Drug Discovery Using Chemical Systems Biology:  Identification of the Protein-Ligand Binding Network to Explain the Side Effects of CETP Inhibitors**

Li Xie, Jerry Li, Lei Xie, Philip E. Bourne


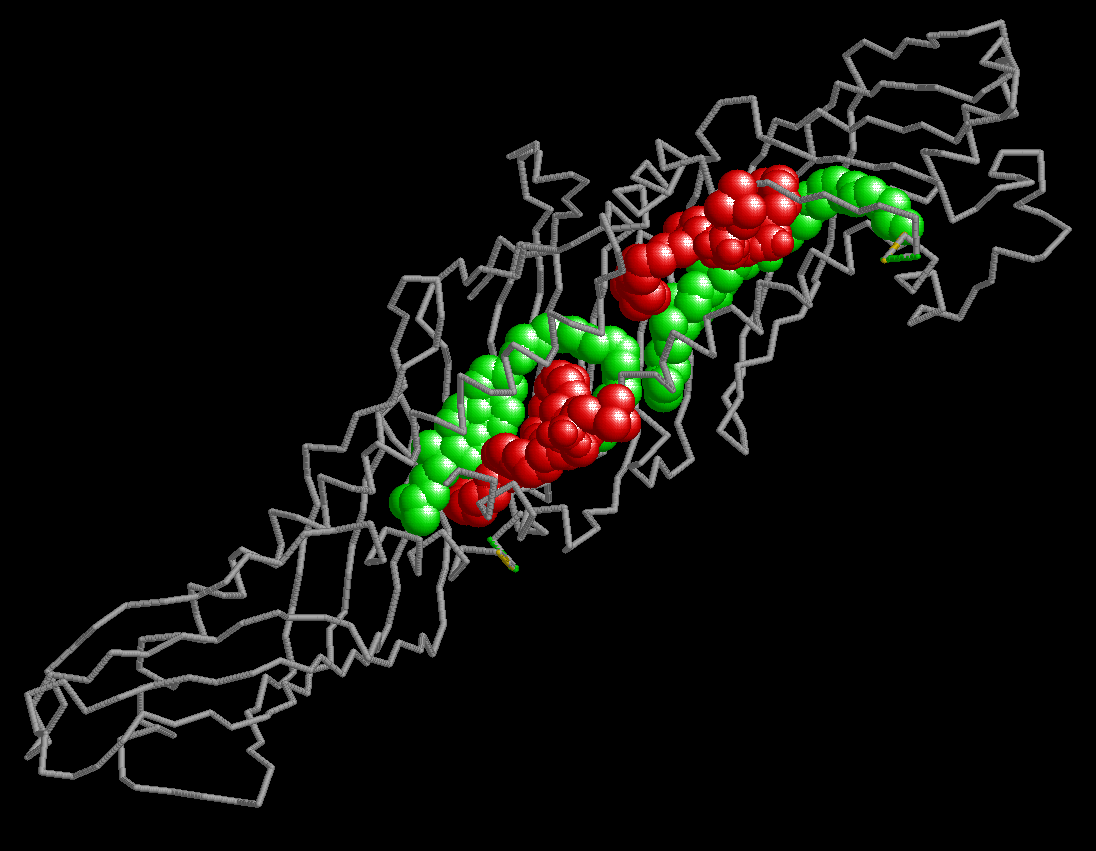


**Figure S1. Four endogenous ligands in the CETP complex structure (PDB id: 2OBD). The two 2OB and two PCW molecules are colored green and red, respectively.**
