# Supplementary material for: Drug Discovery Using Chemical Systems Biology: Identification of the Protein-Ligand Binding Network To Explain the Side Effects of CETP Inhibitors
Source: PLoS Comput Biol. 2009 May 15;5(5):e1000387. doi: 10.1371/journal.pcbi.1000387 (PMC2676506; doi:10.1371/journal.pcbi.1000387)
Supplement: Figure S2 — Structural coverage of the human proteome vs. alignment length between the protein sequence and the structural template. (0.06 MB DOC) [file pcbi.1000387.s002.doc]

**Drug Discovery Using Chemical Systems Biology:  Identification of the Protein-Ligand Binding Network to Explain the Side Effects of CETP Inhibitors**

Li Xie, Jerry Li, Lei Xie, Philip E. Bourne

**
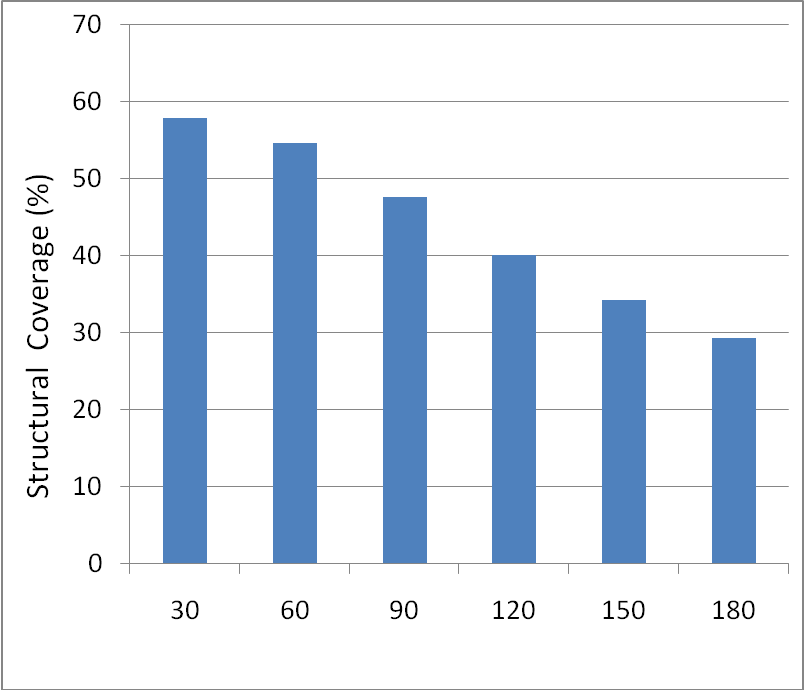
**

**Figure S2. Structural coverage of the human proteome vs. alignment length between the protein sequence and the structural template. The alignment is derived from a PSI-Blast search (e-value < 1.0e-3).**
