# Supplementary material for: Drug Discovery Using Chemical Systems Biology: Identification of the Protein-Ligand Binding Network To Explain the Side Effects of CETP Inhibitors
Source: PLoS Comput Biol. 2009 May 15;5(5):e1000387. doi: 10.1371/journal.pcbi.1000387 (PMC2676506; doi:10.1371/journal.pcbi.1000387)
Supplement: Figure S3 — CE Z-score distributions of putative off-targets. (0.06 MB DOC) [file pcbi.1000387.s003.doc]

**Drug Discovery Using Chemical Systems Biology:  Identification of the Protein-Ligand Binding Network to Explain the Side Effects of CETP Inhibitors**

Li Xie, Jerry Li, Lei Xie, Philip E. Bourne

**
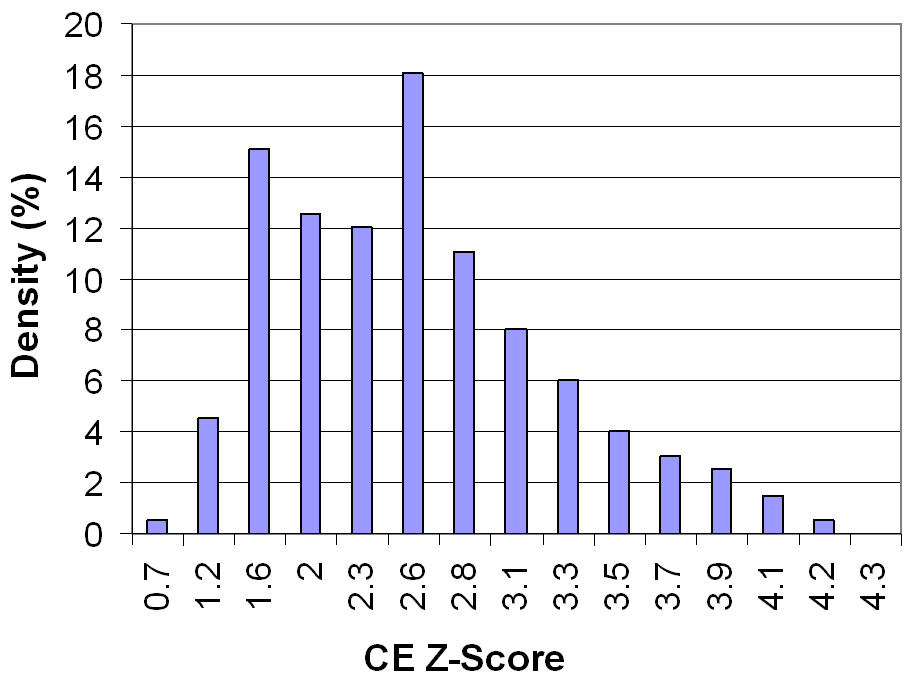
**

**Figure S3. CE Z-score distributions of putative off-targets. Z-scores are usually larger than 3.7 and 4.0 for fold and superfamily level similarity, respectively [1].**

**Reference:**

1. Shindyalov IN, Bourne PE (1998) Protein structure alignment by incremental combinatorial extension (CE) of the optimal path. Protein Engng 9: 739--747.
