# Supplementary material for: Drug Discovery Using Chemical Systems Biology: Identification of the Protein-Ligand Binding Network To Explain the Side Effects of CETP Inhibitors
Source: PLoS Comput Biol. 2009 May 15;5(5):e1000387. doi: 10.1371/journal.pcbi.1000387 (PMC2676506; doi:10.1371/journal.pcbi.1000387)
Supplement: Figure S4 — Structural clusters of helix-like proteins. (0.07 MB DOC) [file pcbi.1000387.s004.doc]

**Drug Discovery Using Chemical Systems Biology:  Identification of the Protein-Ligand Binding Network to Explain the Side Effects of CETP Inhibitors**

Li Xie, Jerry Li, Lei Xie, Philip E. Bourne

**
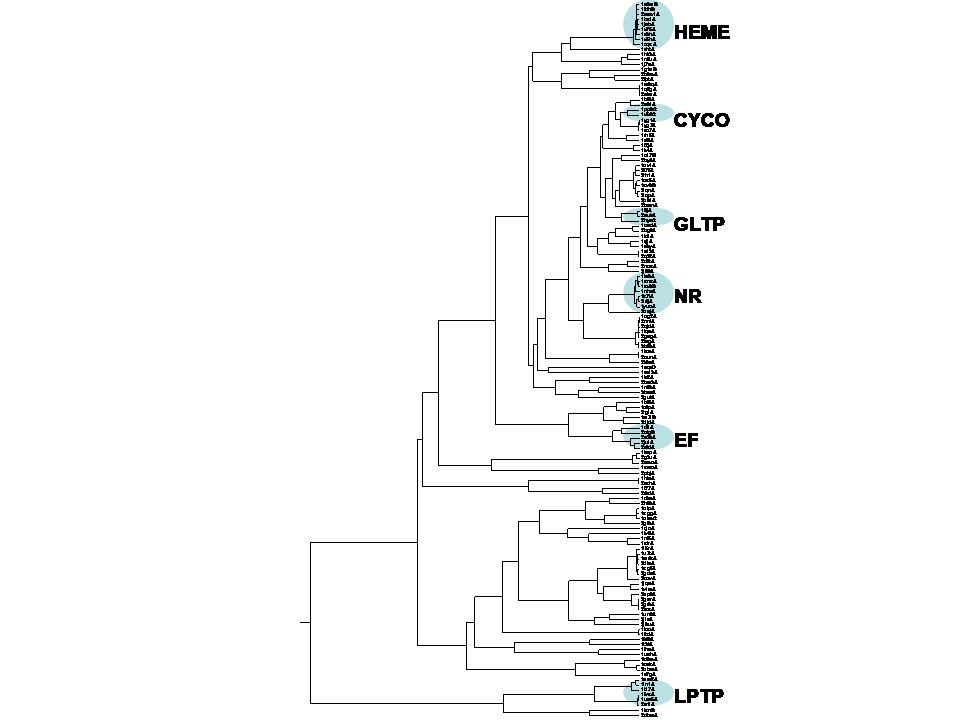
**

**Figure S4. Structural clusters of helix-like proteins. Highlighted clusters are studied in this paper: HEME, Globin-like heme binding protein; CYCO, cytochrome B; GLTP, glycolipid transport protein; NR, nuclear receptor ligand binding domain; EF, EF hand-like calcium binding protein; and LPTP, lipid transport protein.**
