# Supplementary material for: Drug Discovery Using Chemical Systems Biology: Identification of the Protein-Ligand Binding Network To Explain the Side Effects of CETP Inhibitors
Source: PLoS Comput Biol. 2009 May 15;5(5):e1000387. doi: 10.1371/journal.pcbi.1000387 (PMC2676506; doi:10.1371/journal.pcbi.1000387)
Supplement: Figure S5 — Global structure similarity between glycolipid transport protein (PDB: 1tfj) and nuclear hormone receptor ligand binding domain (PDB: 1yow). (0.17 MB DOC) [file pcbi.1000387.s005.doc]

**Drug Discovery Using Chemical Systems Biology:  Identification of the Protein-Ligand Binding Network to Explain the Side Effects of CETP Inhibitors**

Li Xie, Jerry Li, Lei Xie, Philip E. Bourne

**
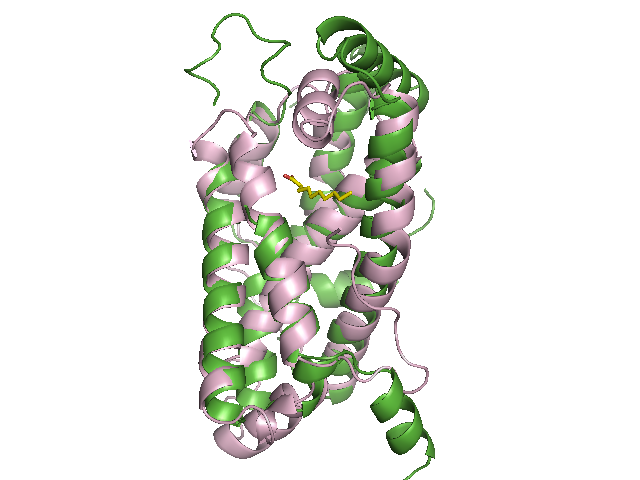
**

**Figure S5. Global structure similarity between glycolipid transport protein (PDB: 1tfj) and nuclear hormone receptor ligand binding domain (PDB: 1yow). The crystal structure of 1tfj and 1yow are colored pink and green, respectively. Yellow sticks represent the ligand (DKA: decanoic acid) of 1tfj.**
