# Supplementary material for: Drug Discovery Using Chemical Systems Biology: Identification of the Protein-Ligand Binding Network To Explain the Side Effects of CETP Inhibitors
Source: PLoS Comput Biol. 2009 May 15;5(5):e1000387. doi: 10.1371/journal.pcbi.1000387 (PMC2676506; doi:10.1371/journal.pcbi.1000387)
Supplement: Figure S6 — Regression curves of eHiTs score for CETP and its off-targets dependent on the number of carbon atoms for a) 2obd, b) 1yow, c) 1y0s, d) 2p54, e) 1zeo, and f) 1ie8. (0.38 MB DOC) [file pcbi.1000387.s006.doc]

**Drug Discovery Using Chemical Systems Biology:  Identification of the Protein-Ligand Binding Network to Explain the Side Effects of CETP Inhibitors**

Li Xie, Jerry Li, Lei Xie, Philip E. Bourne

**
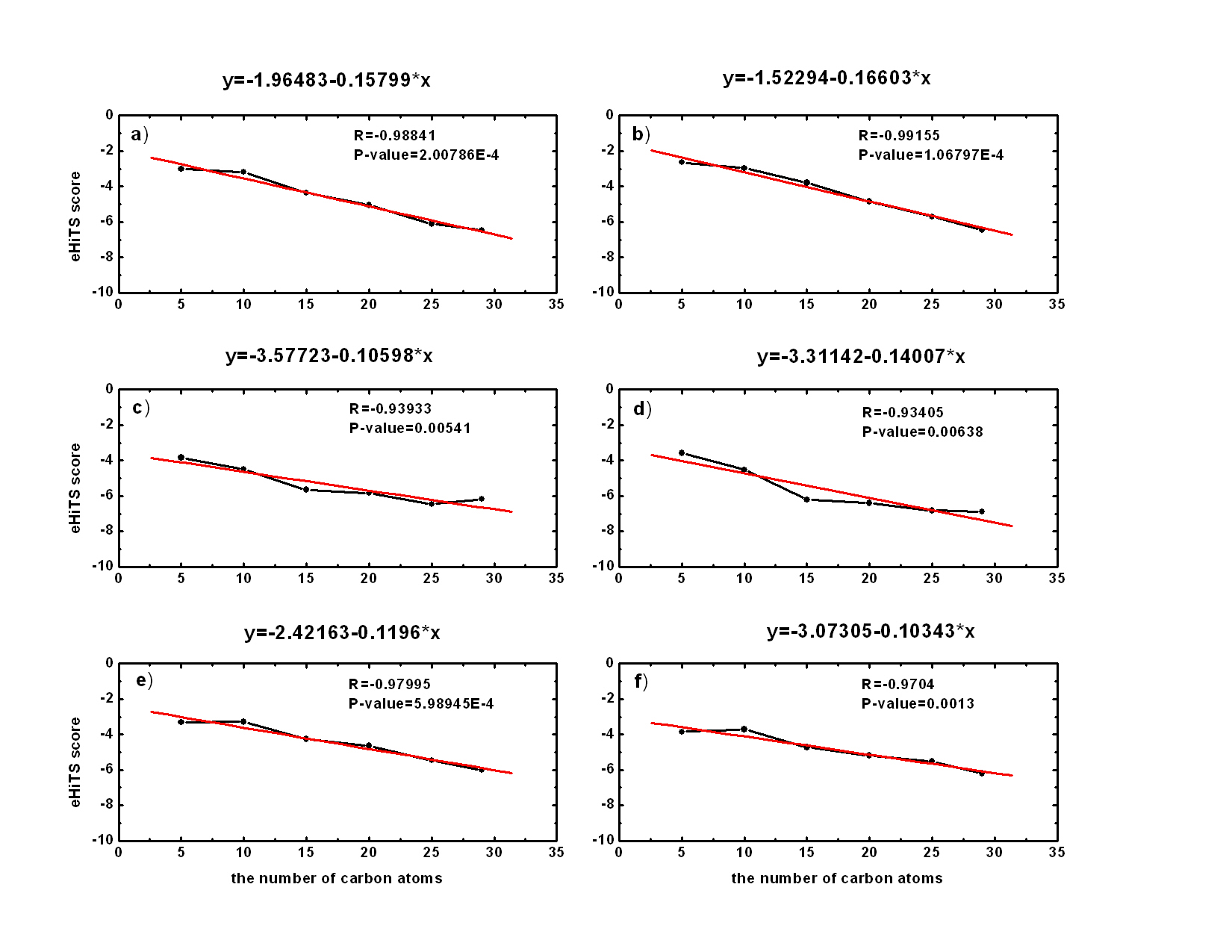
**

**Figure S6. Regression curves of eHiTs score for CETP and its off-targets dependent on the number of carbon atoms for a) 2obd, b) 1yow, c) 1y0s, d) 2p54, e) 1zeo, and f) 1ie8.** **Other proteins studied in this paper show the same trend.**
