# Supplementary material for: Drug Discovery Using Chemical Systems Biology: Identification of the Protein-Ligand Binding Network To Explain the Side Effects of CETP Inhibitors
Source: PLoS Comput Biol. 2009 May 15;5(5):e1000387. doi: 10.1371/journal.pcbi.1000387 (PMC2676506; doi:10.1371/journal.pcbi.1000387)
Supplement: Figure S7 — Correlation of eHiTS score between CETP and its off-targets binding with random ligands with different sizes. a) 1yow; b) 1y0s; c) 2p54; d) 1zeo; e) 1ie8; f) 1tfj. (2.04 MB DOC) [file pcbi.1000387.s007.doc]

**Drug Discovery Using Chemical Systems Biology:  Identification of the Protein-Ligand Binding Network to Explain the Side Effects of CETP Inhibitors**

Li Xie, Jerry Li, Lei Xie, Philip E. Bourne

**
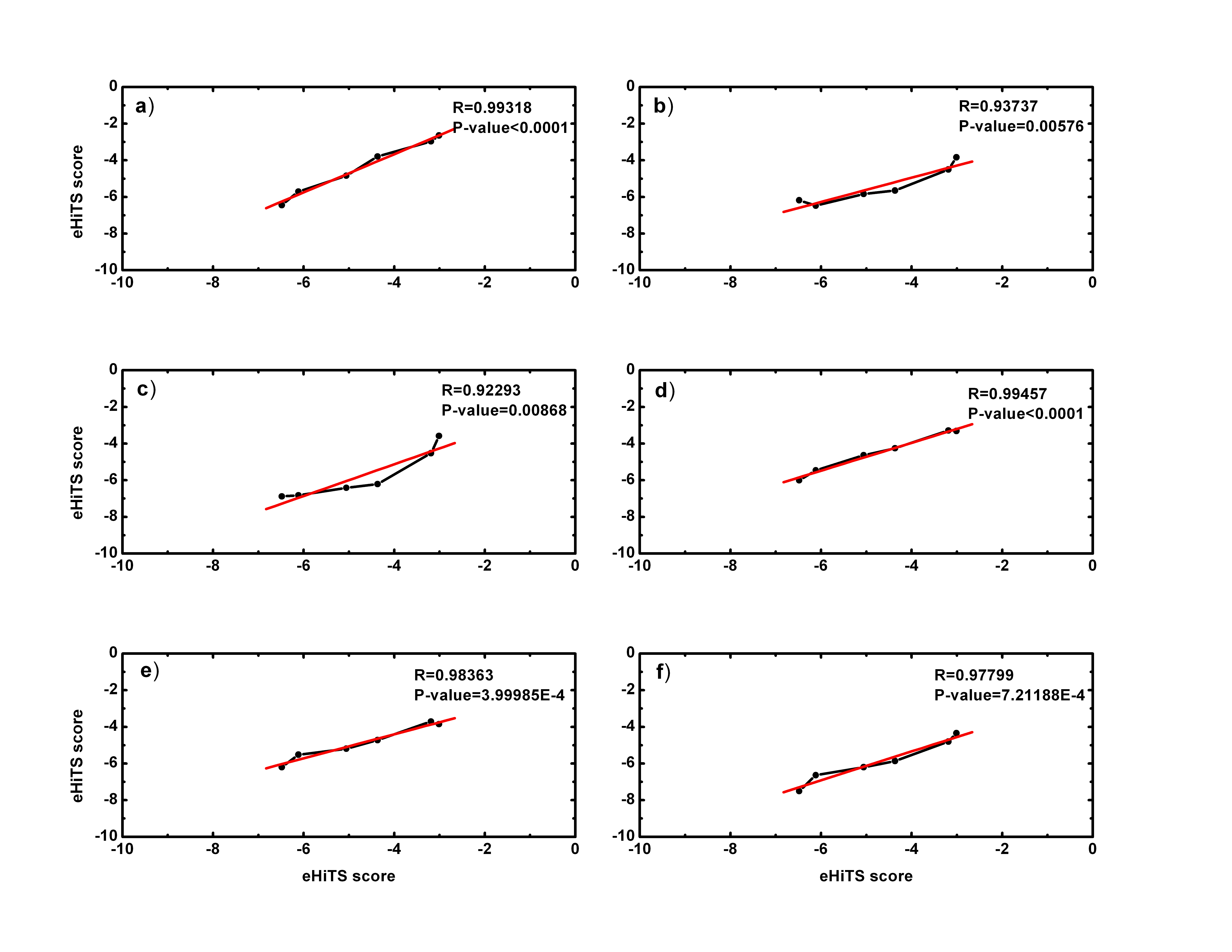
**

**Figure S7. Correlation of eHiTS score between CETP and its off-targets binding with random ligands with different sizes. a) 1yow; b) 1y0s; c) 2p54; d) 1zeo; e) 1ie8; f) 1tfj. Other proteins studied in this paper show the same trend.**
