# Supplementary material for: Drug Discovery Using Chemical Systems Biology: Identification of the Protein-Ligand Binding Network To Explain the Side Effects of CETP Inhibitors
Source: PLoS Comput Biol. 2009 May 15;5(5):e1000387. doi: 10.1371/journal.pcbi.1000387 (PMC2676506; doi:10.1371/journal.pcbi.1000387)
Supplement: Figure S8 — Correlation of the off-target interaction network of CETP inhibitors with the clinical indication through interconnected biological pathways. (0.23 MB DOC) [file pcbi.1000387.s008.doc]

**Drug Discovery Using Chemical Systems Biology:  Identification of the Protein-Ligand Binding Network to Explain the Side Effects of CETP Inhibitors**

Li Xie, Jerry Li, Lei Xie, Philip E. Bourne

**
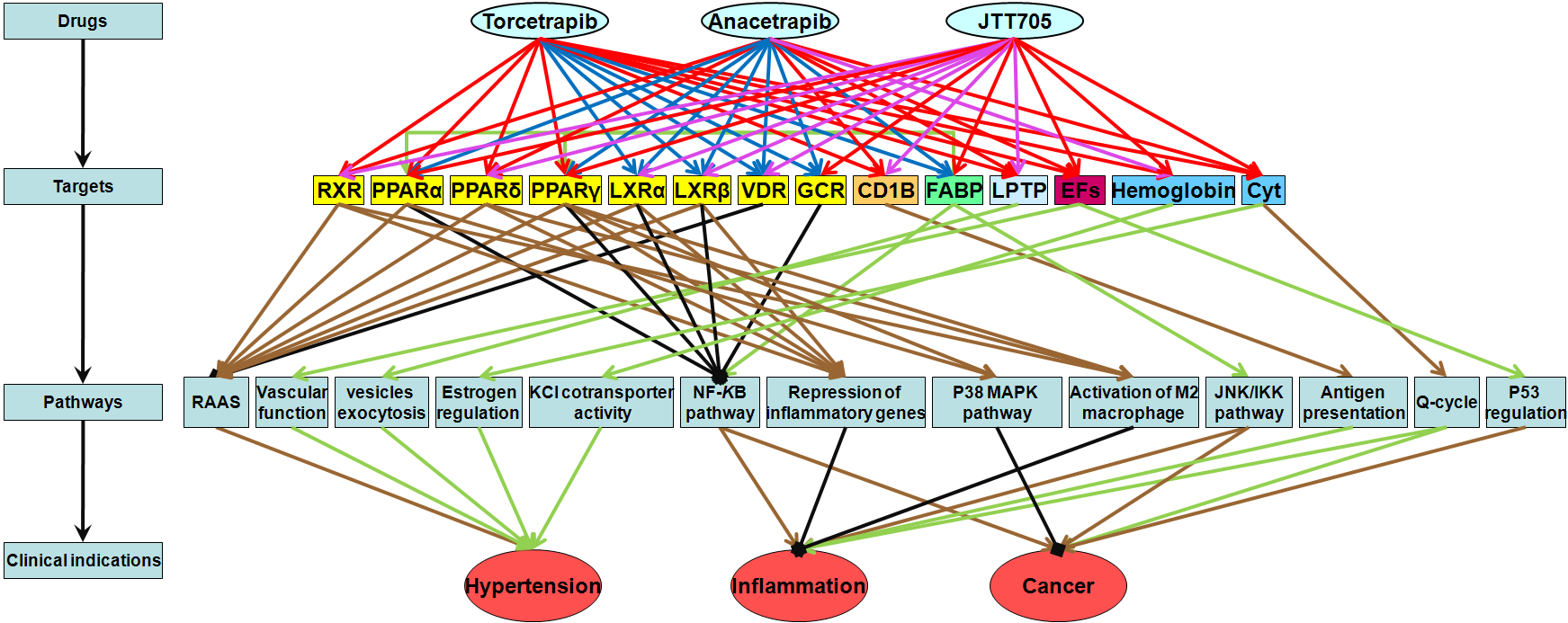
**

**Figure S8.** Correlation of the off-target interaction network of CETP inhibitors with the clinical indication through interconnected biological pathways. The six off-target classes NR, CD1B, FABP, LPTP, EF, and HEME are colored as yellow, orange, green, light blue, purple, and blue, respectively. The red, purple, and blue lines between drugs and targets indicate strong, relatively strong, and weak binding affinity, respectively. The brown and black lines between targets and pathways or clinical indications represent positive and negative regulation, respectively. The green line means positive or negative regulation.
