# Supplementary material for: Drug Discovery Using Chemical Systems Biology: Identification of the Protein-Ligand Binding Network To Explain the Side Effects of CETP Inhibitors
Source: PLoS Comput Biol. 2009 May 15;5(5):e1000387. doi: 10.1371/journal.pcbi.1000387 (PMC2676506; doi:10.1371/journal.pcbi.1000387)
Supplement: Figure S9 — The different regulation effects of Torcetrapib, Anacetrapib and JTT-705 on hypertension, inflammation and cancer through combinational control of other identified off-targets. (0.10 MB DOC) [file pcbi.1000387.s009.doc]

**Drug Discovery Using Chemical Systems Biology:  Identification of the Protein-Ligand Binding Network to Explain the Side Effects of CETP Inhibitors**

Li Xie, Jerry Li, Lei Xie, Philip E. Bourne

**
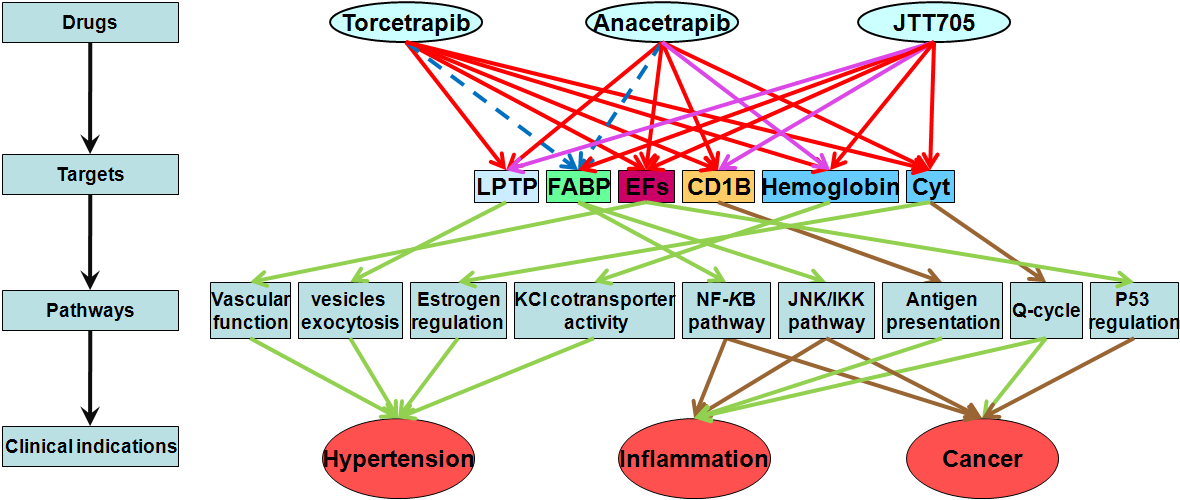
**

**Figure S9.** The Different regulation effects of Torcetrapib, Anacetrapib and JTT-705 on hypertension, inflammation and cancer through combinational control of other identified off-targets. The red, purple, and blue lines between inhibitors and off-targets indicate strong, relatively strong, and weak binding affinity, respectively. The brown lines between off-targets and pathways or clinical indications represent positive and negative regulation, respectively. The green line means positive or negative regulation.
