# Supplementary material for: Drug Discovery Using Chemical Systems Biology: Identification of the Protein-Ligand Binding Network To Explain the Side Effects of CETP Inhibitors
Source: PLoS Comput Biol. 2009 May 15;5(5):e1000387. doi: 10.1371/journal.pcbi.1000387 (PMC2676506; doi:10.1371/journal.pcbi.1000387)
Supplement: Table S1 — Putative off-targets of CETP inhibitors across the human structural genome identified from the off-target pipeline SMAP. (0.05 MB DOC) [file pcbi.1000387.s010.doc]

**Drug Discovery Using Chemical Systems Biology:  Identification of the Protein-Ligand Binding Network to Explain the Side Effects of CETP Inhibitors**

Li Xie, Jerry Li, Lei Xie, Philip E. Bourne

**Table S1. Putative off-targets of CETP inhibitors across the human structural genome identified from the off-target pipeline SMAP.**

**Protein PDB SMAP Surflex eHits**

**Chain e-value score score**

**Septin 7 2QAG_C 5.33E-10 1.58 -4.088**

**CD1 2H26_A 1.30E-9 4.06 -6.164**

**Liver X receptor beta 1UPV_A 4.68E-8 3.09 -4.394**

**CD1 1UQS_A 4.68E-7 2.98 -6.092**

**Septin 6 2QAG_B 5.34E-7 0.69 -0.229**

**Mucin 1 2ACM_A 5.44E-7 1.34 -2.300**

**Glucose 1 dehydrogenase 2DTE_A 6.03E-7 3.33 -0.980**

**Phosphatidylcholine transfer protein 1LN1_A 1.30E-6 1.73 -4.823**

**Glycolipid transfer protein 1TFJ_A 1.68E-6 1.65 -5.595**

**Rho related GTP binding protein RhoC 1Z2C_C 2.12E-6 0.52 -4.426**

**ARC/MEDIATOR, Positive cofactor 2 glutamine/Q rich associate 2GUT_A 2.43E-6 0.34 -3.071**

**RNA Polymerase 2DZ6_A 2.54E-6 2.26 -4.660**

**CD1 1XZ0_A 2.65E-6 2.50 -4.572**

**BCL 2 RELATED PROTEIN A1 2VM6_A 2.83E-6 0.42 -4.704**

**ACETYLCHOLINE RECEPTOR PROTEIN, DELTA CHAIN 2BG9_C 4.82E-6 0.94 -5.057**

**cellular retinaldehyde binding protein model 1XGG_A 5.51E-6 3.07 -6.433**

**Toll like receptor 2 2Z7X_A 5.73E-6 2.57 -6.053**

**INTERLEUKIN 10 1INR_A 6.26E-6 1.59 -5.746**

**MYOSIN LIGHT CHAIN 1 1W7I_B 9.23E-6 1.57 -4.400**

**Skeletal muscle Actin 1M8Q_A 1.01E-5 2.63 -4.908**

**Alpha 2 macroglobulin receptor associated protein 2P01_A 1.08E-5 2.67 -2.065**

**STERYL SULFATASE 1P49_A 1.17E-5 1.48 -4.130**

**GPCR model 2IQP_A 1.32E-5 0.91 -4.366**

**Undecaprenyl Pyrophosphate Synthase model 2H69_A 1.82E-5 2.14 -6.262**

**S100B 1CFP_A 1.99E-5 0.01 -3.160**

**L Gulonate 3 Dehydrogenase 2EP9_A 2.24E-5 1.72 -2.488**

**facilitative glucose transporter model 1YG1_A 2.28E-5 1.62 -3.021**

**PROTEIN (HISTONE H4)(Gallus gallus)|PROTEIN (HISTONE H3)(Gal 1EQZ_D 2.58E-5 0.23 -2.322**

**Globin like 1CQX_A 2.70E-5 3.13 -3.796**

**Phosphatidylinositol transfer protein 2A1L_A 2.76E-5 1.31 -3.289**

**Glycolipid transfer protein 2EUK_A 2.96E-5 2.78 -6.154**

**ACETYLCHOLINE RECEPTOR PROTEIN, GAMMA CHAIN 2BG9_E 3.27E-5 3.39 -3.020**

**CD1 1ZT4_A 3.53E-5 1.15 -4.154**

**Calsenilin 2JUL_A 3.95E-5 0.53 -5.069**

**Glutathione S transferase A3 3 1TDI_A 4.43E-5 2.25 -4.437**

**Plasmodium falciparum hexose transporter model 1LVI_A 4.61E-5 0.40 -3.750**

**PYRROLINE 5 CARBOXYLATE REDUCTASE 1 2IZZ_A 4.68E-5 1.53 -3.079**

**Cytochrome P450 2NNI_A 4.74E-5 1.66 -3.236**

**SARCOPLASMIC/ENDOPLASMIC RETICULUM CALCIUM ATPASE 1 2BY4_A 4.78E-5 1.96 -4.995**

**Bile acid receptor 3BEJ_A 5.00E-5 3.17 -5.676**

**Cytochrome P450 1OG2_A 5.08E-5 2.38 -2.272**

**Bifunctional coenzyme A synthase 2F6R_A 5.12E-5 2.48 -2.394**

**SAM dependent methyltransferase 1VLM_A 5.41E-5 1.32 -1.647**

**Globin like 1BZ1_A 5.51E-5 1.73 -5.381**

**Hemoglobin D alpha chain 1V75_A 5.60E-5 0.65 -7.217**

**Steroidogenic factor 1 1YOW_A 6.15E-5 3.83 -0.391**

**DIHYDROXYACETONE KINASE C 1UN9_A 6.32E-5 0.90 -7.844**

**Endothelial protein C receptor 1L8J_A 6.37E-5 0.85 -6.477**

**serum albumin 1N5U_A 6.53E-5 4.16 -3.417**

**Lymphocyte antigen 96 (MD 2) 2Z64_B 6.57E-5 1.94 -5.779**

**ACETYLCHOLINE RECEPTOR PROTEIN, GAMMA CHAIN 1OED_A 6.57E-5 0.20 -1.859**

**Retinoic acid receptor RXR beta 1UHL_A 6.68E-5 1.21 -6.282**

**APOCAROTENOID CLEAVING OXYGENASE 2BIW_A 6.98E-5 3.06 -3.362**

**Voltage Gated Sodium Channel model 2DJD_A 8.04E-5 2.43 -2.011**

**L LACTATE DEHYDROGENASE 1LCO_A 8.45E-5 1.52 -4.861**

**AHCPK2 model 2G3U_A 8.54E-5 2.05 -4.956**

**Cardiac troponin C 1DTL_A 8.78E-5 1.68 -8.538**

**Calsenilin 2E30_A 9.04E-5 3.83 -3.570**

**Cytochrome B 1PP9_C 9.19E-5 3.40 -6.573**

**Crossover junction endonuclease MUS81 2ZIX_A 9.30E-5 2.22 -4.564**

**ALPHA 1 ANTICHYMOTRYPSIN 2ACH_A 9.35E-5 0.83 -4.763**

**GM2 activator protein 1PUB_A 9.41E-5 2.71 -2.535**

**BACTERIAL DYNAMIN LIKE PROTEIN 2J68_A 9.46E-5 2.64 -4.472**

**facilitative glucose transporter model 1YG7_A 9.80E-5 2.84 -2.168**

**GPCR model 1OZ5_A 9.94E-5 0.47 -3.391**

**Pyrroline 5 carboxylate reductase 1 2GR9_A 1.02E-4 1.54 -2.753**

**SAPOSIN B 1N69_A 1.04E-4 0.25 -6.723**

**6 phosphofructo 2 kinase 1K6M_A 1.08E-4 1.72 -2.685**

**Globin like 2MM1_A 1.09E-4 1.12 -6.316**

**NAD dependent deacetylase 2 1S7G_A 1.09E-4 2.60 -2.053**

**Prostacyclin synthase 2IAG_A 1.10E-4 1.84 -0.332**

**Tryptophan 2,3 dioxygenase(Ralstonia metallidurans) 2NOX_A 1.18E-4 3.21 -3.421**

**Nuclear receptor ligand binding domain 1YUC_A 1.19E-4 3.63 -5.420**

**Vitamin D binding protein 1J7E_A 1.21E-4 0.75 -3.255**

**ATP SYNTHASE SUBUNIT A(Escherichia coli) 1C17_M 1.25E-4 2.25 -3.802**

**ACETYLCHOLINE RECEPTOR PROTEIN, GAMMA CHAIN 2BG9_A 1.32E-4 3.10 -5.561**

**PROTEIN (CELLULAR RETINOL BINDING PROTEIN III) 1GGL_A 1.34E-4 3.10 -0.375**

**Beta Platelet Derived Growth Factor Receptor model 1LWP_A 1.36E-4 1.92 -2.690**

**Nuclear receptor ligand binding domain 1M13_A 1.38E-4 2.97 -6.448**

**Interferon gamma 3BES_A 1.38E-4 2.06 -4.681**

**Heme oxygenase like 2Q32_A 1.40E-4 1.92 -3.958**

**progesterone receptor 1SQN_A 1.43E-4 2.18 -3.433**

**Polyketide cyclase/dehydrase and lipid transport protein 1T17_A 1.44E-4 3.11 -4.019**

**FLAVOCYTOCHROME B2 1LTD_A 1.44E-4 2.14 -3.790**

**Phosphatidylinositol transfer protein 1FVZ_A 1.44E-4 3.81 -3.444**

**CYCLOOXYGENASE 2 1CX2_A 1.48E-4 1.52 -2.519**

**Nuclear receptor ligand binding domain 1IE8_A 1.55E-4 1.46 -4.586**

**Dual specificity protein kinase TTK 2ZMC_A 1.56E-4 1.04 -5.441**

**Resistin model 1LV6_A 1.59E-4 0.46 -3.741**

**somatostatin model 1P2W_A 1.61E-4 1.57 -4.979**

**Dynein Motor Unit model 2GF8_A 1.67E-4 1.52 -2.270**

**SIN3A(Mus musculus) 1G1E_B 1.72E-4 2.24 -2.205**

**SEC14 LIKE PROTEIN 2 1OLM_C 1.74E-4 0.69 -3.344**

**Globin like 1S0H_A 1.77E-4 2.05 -6.827**

**facilitative glucose transporter model 1I2J_A 1.80E-4 2.80 -2.861**

**KETOACYL REDUCTASE 1W4Z_A 1.87E-4 1.73 -0.097**

**MITOCHONDRIAL CARNITINE ACYLCARNITINE TRANSPORTER model 2BMN_A 1.94E-4 0.69 -2.293**

**Nuclear receptor ligand binding domain 1XNX_A 1.97E-4 3.29 -1.850**

**Cytochrome P450 2GEG_A 2.01E-4 2.45 -3.531**

**PPAR delta 2J14_A 2.06E-4 0.23 -5.356**

**HISTIDINE AMMONIA LYASE 1B8F_A 2.08E-4 1.46 -2.730**

**Globin like 1JEB_A 2.26E-4 0.04 -5.715**

**Fatty acid binding protein, liver 2PY1_A 2.27E-4 3.42 -4.661**

**Beta integrin model 1LHA_A 2.28E-4 1.54 -1.054**

**EF hand like 2RGI_A 2.30E-4 0.89 -3.318**

**EPOXIDE HYDROLASE 1EK2_A 2.31E-4 2.20 -5.364**

**3',5' cyclic nucleotide phosphodiesterase 1LXS_A 2.37E-4 0.60 -5.610**

**EF hand like 1BT6_A 2.41E-4 0.39 -1.866**

**polymerase (DNA directed) iota 1T3N_C 2.55E-4 1.32 -3.193**

**Monocyte differentiation antigen CD14 1WWL_A 2.61E-4 1.90 -6.353**

**GPCR model 1OV1_A 2.88E-4 1.27 -3.587**

**HORSE LEUKOCYTE ELASTASE INHIBITOR 1HLE_A 3.21E-4 0.74 -1.845**

**GPCR model 2IQN_A 3.30E-4 3.16 -4.730**

**Phosphoribosylformylglycinamidine synthase 1T3T_A 3.31E-4 0.43 -0.373**

**ADENYLATE KINASE 1ZAK_A 3.39E-4 2.57 -3.867**

**Globin like 1V5H_A 3.62E-4 6.56 -3.756**

**serum albumin 1HK3_A 3.65E-4 1.74 -4.110**

**CELLULAR RETINOL BINDING PROTEIN III 1GGL_A 3.70E-4 3.15 -0.096**

**Phosphatidylinositol transfer protein 1UW5_A 3.78E-4 1.42 -4.441**

**STAR related lipid transport domain of MLN64 1EM2_A 3.94E-4 1.17 -0.499**

**ACETYLCHOLINE RECEPTOR PROTEIN, BETA CHAIN 2BG9_B 3.96E-4 2.36 -5.745**

**DC SIGNR ectodomain 1Z0Y_A 4.03E-4 1.20 -1.397**

**Dihydroorotate dehydrogenase, mitochondrial 2FPT_A 4.05E-4 2.47 -6.454**

**actinin(Gallus gallus) 1SJJ_A 4.09E-4 0.11 -1.042**

**GPCR model 1ZV0_B 4.21E-4 2.67 -0.258**

**Prostaglandin E synthase 2 2PBJ_A 4.31E-4 0.65 -4.322**

**Indoleamine 2,3 dioxygenase 2D0T_A 4.43E-4 2.90 -3.976**

**PROTEIN (RETINOID X RECEPTOR ALPHA) 1DKF_A 4.46E-4 3.89 -6.086**

**Gap junction beta 2 protein 1XIR_A 4.53E-4 0.45 -2.559**

**UTP-glucose 1 phosphate uridylyltransferase 2I5K_A 4.54E-4 1.85 -5.566**

**Nuclear receptor ligand binding domain 1K7L_A 4.61E-4 0.40 -3.137**

**NAD+ dependent 15 hydroxyprostaglandin dehydrogenase 2GDZ_A 4.62E-4 0.54 -1.060**

**CYSTEINE SULFINIC ACID DECARBOXYLASE 2JIS_A 4.64E-4 1.00 -3.167**

**Preprotein translocase secY subunit 1RH5_A 4.66E-4 2.37 -5.146**

**CAMP BINDING DOMAINS OF CAMP DEPENDENT PROTEIN KINASE 1APK_A 4.74E-4 0.21 -2.314**

**Gamma glutamyltranspeptidase 2DBW_A 4.89E-4 0.91 -2.535**

**TRANSPOSASE 2BW3_A 5.03E-4 0.83 -4.024**

**Nuclear receptor ligand binding domain 2I4J_A 5.08E-4 4.57 -5.987**

**quinone oxidoreductase 2ZCV_A 5.19E-4 2.19 -1.214**

**ATP DEPENDENT RNA HELICASE DDX48 2J0U_A 5.23E-4 0.89 -1.504**

**Apolipoprotein A I 2A01_A 5.52E-4 2.42 -3.244**

**ADRENODOXIN REDUCTASE 1CJC_A 5.54E-4 1.94 -4.743**

**Oxysterols receptor LXR alpha 1UHL_B 5.81E-4 1.71 -5.377**

**Globin like 1A9W_B 5.82E-4 0.51 -5.011**

**Pyrroline 5 carboxylate reductase 1 2GER_A 5.92E-4 1.17 -2.644**

**ALPHA TOCOPHEROL TRANSFER PROTEIN 1OIP_A 5.92E-4 2.75 -4.956**

**Cytochrome P450 aromatase 1TQA_A 5.97E-4 1.51 -2.249**

**PROTOHEME FERROLYASE 1C9E_A 6.00E-4 1.97 -2.369**

**CapZ beta 1 subunit 1IZN_B 6.02E-4 0.73 -2.135**

**KES1, Oxysterol binding protein 1ZHT_A 6.03E-4 0.64 -6.828**

**Prostaglandin I2 synthase 3B98_A 6.10E-4 1.30 -2.800**

**Vinculin(Gallus gallus) 1ST6_A 6.20E-4 0.49 -0.720**

**Mature alpha chain of major histocompatibility complex class 1KJV_A 6.23E-4 1.55 -2.797**

**Myosin light chain 2OTG_B 6.24E-4 1.31 -3.725**

**TYPE 1 17 BETA HYDROXYSTEROID DEHYDROGENASE 1I5R_A 6.36E-4 1.17 -3.486**

**Putative cell cycle protein mesJ(Escherichia coli) 1NI5_A 6.37E-4 1.05 -2.348**

**Cytohesin 3 2R0D_A 6.39E-4 2.07 -2.950**

**BAND 3 ANION TRANSPORT PROTEIN 1HYN_A 6.52E-4 2.27 -3.470**

**SMALL HEAT SHOCK PROTEIN 2BOL_A 6.55E-4 1.41 -3.161**

**Retinol binding protein IV, cellular 1LPJ_A 6.57E-4 0.61 -0.553**

**ESTRADIOL 17 BETA DEHYDROGENASE 4 1IKT_A 6.57E-4 1.79 -3.809**

**Lymphocyte antigen 96 (MD 2) 2E59_A 6.70E-4 1.78 -6.036**

**Hypothetical protein AQ1575 1LFP_A 6.79E-4 2.10 -1.702**

**HLA class I histocompatibility antigen 1KPR_A 6.94E-4 3.61 -2.770**

**HLA class I histocompatibility antigen, B 8 B*0801 alpha chain 1M05_A 6.99E-4 1.98 -0.572**

**3',5' cyclic nucleotide phosphodiesterase 2OUN_A 7.08E-4 1.35 -3.034**

**acyl CoA oxidase(Rattus norvegicus) 1IS2_A 7.17E-4 1.87 -3.366**

**Lipopolysaccharide responsive and beige like anchor protein 1T77_A 7.22E-4 0.38 -2.453**

**Cytochrome P450 1R9O_A 7.30E-4 2.12 -6.151**

**Prostaglandin G/H synthase 1 precursor 1U67_A 7.31E-4 1.42 -3.009**

**ARSENITE TRANSLOCATING ATPASE 1F48_A 7.35E-4 0.90 -1.429**

**Dihydroorotate dehydrogenase, mitochondrial 2B0M_A 7.49E-4 0.58 -3.266**

**Histamine N Methyltransferase 1JQE_A 7.50E-4 1.20 -4.391**

**CYANATE HYDRATASE 2IUO_A 7.51E-4 1.03 -3.440**

**Insulin receptor 2DTG_G 7.56E-4 2.03 -3.714**

**Eukaryotic translation initiation factor 5 2G2K_A 7.71E-4 1.28 -4.290**

**Myosin II heavy chain 2AKA_A 7.87E-4 1.73 -3.347**

**Myosin II motor domain model 1Q5G_A 7.87E-4 1.73 -3.347**

**Nuclear receptor ligand binding domain 1XV9_B 7.91E-4 2.60 -3.544**

**adenylate kinase 4, AK4 2BBW_A 7.93E-4 2.73 -2.173**

**ADP ribosylation factor like protein 2 binding protein 2K0S_A 7.96E-4 2.21 -1.861**

**HLA class I histocompatibility antigen 2ESV_A 7.99E-4 1.39 -3.719**

**beta 2 adrenergic receptor 2RH1_A 8.02E-4 1.98 -3.265**

**Dihydrolipoyllysine residue acetyltransferase 3B8K_A 8.04E-4 0.65 -3.023**

**Oxalate transporter model 1ZC7_A 8.10E-4 1.55 -4.028**

**Cullin 4A 2HYE_C 8.22E-4 0.14 -4.963**

**NECAP1(Mus musculus), unknown function 1TQZ_A 8.23E-4 1.46 -6.281**

**GLUCOCORTICOID RECEPTOR 1NHZ_A 8.36E-4 0.36 -5.788**

**NKG2 D TYPE II INTEGRAL MEMBRANE PROTEIN 1HYR_C 8.37E-4 0.19 -2.072**

**Gastrotropin, Ileal Lipid (Fatty acid) binding protein 1O1V_A 8.40E-4 3.58 -2.462**

**Heme oxygenase like 1S13_A 8.46E-4 2.16 -4.096**

**Proto oncogene tyrosine protein kinase ABL1 2E2B_A 8.65E-4 0.93 -2.716**

**Calsenilin 2ZFD_A 8.79E-4 3.91 -2.032**

**Cytochrome c oxidase polypeptide III 1V54_C 8.83E-4 0.89 -5.109**

**3 hydroxyacyl CoA dehydrogenase type II 1U7T_A 8.99E-4 0.84 -3.996**

**Peroxisomal carnitine O octanoyltransferase 1XMC_A 9.05E-4 3.05 -5.017**

**50S ribosomal protein L14 1VS9_I 9.06E-4 2.65 -1.726**

**GPCR model 2F75_A 9.09E-4 1.19 -3.822**

**HLA class I histocompatibility antigen 2BCK_A 9.13E-4 2.27 -3.138**

**Acyl CoA N acyltransferases 1KUX_A 9.14E-4 0.64 -1.131**

**Globin like 1FDH_B 9.24E-4 1.16 -4.440**

**DADP DEPENDENT ALDEHYDE DEHYDROGENASE 1UEH_A 9.66E-4 3.45 -5.590**

**PROTOPORPHYRINOGEN OXIDASE(Myxococcus xanthus) 2IVD_A 9.80E-4 2.72 -2.739**

**putative dehydrogenase 1XG5_A 9.92E-4 1.20 -2.618**

**Cytochrome P450 2OJD_A 9.93E-4 1.16 -6.070**
