# Supplementary material for: Drug Discovery Using Chemical Systems Biology: Identification of the Protein-Ligand Binding Network To Explain the Side Effects of CETP Inhibitors
Source: PLoS Comput Biol. 2009 May 15;5(5):e1000387. doi: 10.1371/journal.pcbi.1000387 (PMC2676506; doi:10.1371/journal.pcbi.1000387)
Supplement: Table S2 — GO based similarity between CETP and off-targets. (0.03 MB DOC) [file pcbi.1000387.s011.doc]

**Drug Discovery Using Chemical Systems Biology:  Identification of the Protein-Ligand Binding Network to Explain the Side Effects of CETP Inhibitors**

Li Xie, Jerry Li, Lei Xie, Philip E. Bourne

**Table S2. GO based similarity between CETP and off-targets. Here Resnik scores [1], which are evaluated as the best in the benchmark study [2], are used to measure the similarity.**

**Protein PDB Max Avg**

Oxysterol binding protein 1ZHT_A 2.96 2.96

STAR related lipid transport domain of MLN64 EM2_A 2.96 2.96

Gastrotropin, Ileal Lipid (Fatty acid) binding protein 1O1V_A 2.96 2.96

Cytohesin 3 2R0D_A 2.96 1.66

Vitamin D binding protein 1J7E_A 2.96 1.66

Glycolipid transfer protein 1TFJ_A 2.96 1.48

SAPOSIN B(Homo sapiens) 1N69_A 2.96 1.48

SEC14 like protein 2 1OLM_C 2.96 1.10

Apolipoprotein A I(Homo sapiens) 2A01_A 2.96 1.10

T-cell surface glycoprotein CD1d 1ZT4_A 2.96 0.91

Nuclear receptor ligand binding domain 2J14_A 2.96 0.83

Estradiol 17beta dehydrogenase 1IKT_A 2.96 0.83

Serum albumin 1N5U_A 2.96 0.63

Phosphatidylinositol transfer protein 1UW5_A 0.35 0.12

Globin like protein 1BZ1_A 0.35 0.35

Calsenilin (EF hand-like) 2ZFD_A 0.35 0.35

Transposase 2BW3_A 0.35 0.35

Mature alpha chain of major histocompatibility complex class 1KJV_A 0.35 0.35

Toll like receptor 2 2Z7X_A 0.35 0.35

Myosin light chain 2OTG_B 0.35 0.35

Skeletal muscle Actin(Oryctolagus cuniculus)|Skeletal muscle 1M8Q_A 0.35 0.35

BCL 2 RELATED PROTEIN A1 2VM6_A 0.35 0.35

Bile acid receptor 3BEJ_A 0.35 0.35

Rho related GTP binding protein RhoC(Homo sapiens) 1Z2C_C 0.35 0.35

NECAP1(Mus musculus), unknown function 1TQZ_A 0.35 0.35

Interferon gamma 3BES_A 0.35 0.35

ATP DEPENDENT RNA HELICASE DDX48(Homo sapiens) 2J0U_A 0.35 0.35

Myosin II heavy chain 2AKA_A 0.35 0.35

ARC/MEDIATOR, Positive cofactor 2 glutamine/Q rich associate 2GUT_A 0.35 0.35

ALPHA TOCOPHEROL TRANSFER PROTEIN(Homo sapiens) 1OIP_A 0.35 0.35

HISTONE H4 1EQZ_D 0.35 0.35

Lipopolysaccharide responsive and beige like anchor protein 1T77_A 0.35 0.35

Cullin 4A(Homo sapiens) 2HYE_C 0.35 0.35

PROTEIN (CELLULAR RETINOL BINDING PROTEIN III)(Homo sapiens) 1GGL_A 0.35 0.35

Lymphocyte antigen 96 (MD 2) 2Z64_B 0.35 0.35

INTERLEUKIN 10(Homo sapiens) 1INR_A 0.35 0.35

SIN3A(Mus musculus) 1G1E_B 0.35 0.35

Alpha 2 macroglobulin receptor associated protein 2P01_A 0.35 0.29

Bile acid receptor 3BEJ_A 0.35 0.28

ATP DEPENDENT RNA HELICASE DDX48(Homo sapiens) 2J0U_A 0.35 0.26

PROTEIN (ADRENODOXIN REDUCTASE) 1CJC_A 0.35 0.26

beta 2 adrenergic receptor 2RH1_A 0.35 0.23

progesterone receptor 1SQN_A 0.35 0.23

Steroidogenic factor 1(Homo sapiens) 1YOW_A 0.35 0.23

SARCOPLASMIC/ENDOPLASMIC RETICULUM CALCIUM ATPASE 1 2BY4_A 0.35 0.23

Myosin light chain 2OTG_B 0.35 0.23

ALPHA 1 ANTICHYMOTRYPSIN 2ACH_A 0.35 0.23

Insulin receptor 2DTG_G 0.35 0.22

PROTEIN Vinculin 1ST6_A 0.35 0.17

ACETYLCHOLINE RECEPTOR PROTEIN 1OED_A 0.35 0.17

Phosphoribosylformylglycinamidine synthase 1T3T_A 0.35 0.17

UTP glucose 1 phosphate uridylyltransferase 2I5K_A 0.35 0.17

Glucose 1 dehydrogenase 2DTE_A 0.35 0.17

Proto oncogene tyrosine protein kinase ABL1 2E2B_A 0.35 0.17

NAD+ dependent 15 hydroxyprostaglandin dehydrogenase 2GDZ_A 0.35 0.17

NKG2 D TYPE II INTEGRAL MEMBRANE PROTEIN 1HYR_C 0.35 0.17

BAND 3 ANION TRANSPORT PROTEIN(Homo sapiens) 1HYN_A 0.35 0.17

Eukaryotic translation initiation factor 5(Homo sapiens) 2G2K_A 0.35 0.17

3 hydroxyacyl CoA dehydrogenase type II(Homo sapiens) 1U7T_A 0.35 0.17

BACTERIAL DYNAMIN LIKE PROTEIN(NOSTOC PUNCTIFORME) 2J68_A 0.35 0.17

MYOSIN LIGHT CHAIN 1, 1W7I_B 0.35 0.12

Rho related GTP binding protein RhoC(Homo sapiens) 1Z2C_C 0.35 0.12

6 phosphofructo 2 kinase 1K6M_A 0.35 0.12

**References:**

1. Resnik P (1999) Semantic similarity in a taxonomy: an information-based measure and its application to problems of ambiguity in natural language. Artificial Intelligence Res 11: 95-130.

2. Pesquita C, Faria D, Bastos H, Ferreira AE, Falcao AO, et al. (2008) Metrics for GO based protein semantic similarity: a systematic evaluation. BMC Bioinformatics 9 Suppl 5: S4.
