# Supplementary material for: Drug Discovery Using Chemical Systems Biology: Identification of the Protein-Ligand Binding Network To Explain the Side Effects of CETP Inhibitors
Source: PLoS Comput Biol. 2009 May 15;5(5):e1000387. doi: 10.1371/journal.pcbi.1000387 (PMC2676506; doi:10.1371/journal.pcbi.1000387)
Supplement: Table S3 — Vector distances and Pearson correlations of carbon atom dependent average eHiTS docking scores between binding pockets of CETP and six classes of off-targets. (0.05 MB DOC) [file pcbi.1000387.s012.doc]

**Drug Discovery Using Chemical Systems Biology:  Identification of the Protein-Ligand Binding Network to Explain the Side Effects of CETP Inhibitors**

Li Xie, Jerry Li, Lei Xie, Philip E. Bourne

| **Table S3, Vector distances and Pearson correlations of carbon atom dependent average eHiTS docking scores between binding pockets of CETP and six classes of off-targets. The correlation curves for several proteins are shown in Figure S8.** | | | | | |
| --- | --- | --- | --- | --- | --- |
| Target Class | Protein | PDB ID | Distance | Pearson correlation | P-value |
| NR | Retinoid X receptor (agonist) | 1YOW | 0.3473 | 0.9932 | 0.0000 |
|  | PPARδ (agonist) | 1Y0S | 0.9019 | 0.9374 | 0.0001 |
|  | PPARα (agonist) | 2P54 | 1.1552 | 0.9229 | 0.0003 |
|  | PPARγ (agonist) | 1ZEO | 0.3956 | 0.9946 | 0.0000 |
|  | LXRα (agonist) | 2ACL | 0.2599 | 0.9953 | 0.0000 |
|  | LXRβ (agonist) | 1UPV | 0.7162 | 0.9957 | 0.0000 |
|  | Vitamin D receptor (agonist) | 1IE8 | 0.5101 | 0.9836 | 0.0000 |
|  | Glucocorticoid receptor (agonist) | 1P93 | 0.4719 | 0.9929 | 0.0000 |
|  | Glucocorticoid receptor (antagonist) | 1NHZ | 0.5608 | 0.9479 | 0.0001 |
| LPTP | Glycolipid transfer protein | 1TFJ | 1.2428 | 0.9780 | 0.0000 |
|  | Phosphatidylcholine transfer protein | 1LN1 | 0.5327 | 0.9850 | 0.0000 |
|  | Phosphatidylinositol transfer protein | 2A1L | 0.4095 | 0.9690 | 0.0000 |
|  | GM-2 activator | 2AG9 | 0.6116 | 0.9839 | 0.0000 |
| FABP | A-Fatty acid bindind protein | 2NNQ | 1.6013 | 0.9234 | 0.0003 |
| CD1B | T-cell CD1B | 1GZP | 0.6651 | 0.9919 | 0.0000 |
| EF | **Troponin C** | 1DTL | 0.8397 | 0.9970 | 0.0000 |
| HEME | Cytochrome complex | 1PP9 | 0.4751 | 0.9661 | 0.0000 |
|  | Human cytoglobin | 1V5H | 0.6185 | 0.9710 | 0.0000 |
